# LCMS/MS Phytochemical Profiling, Molecular, Pathological, and Immune-Histochemical Studies on the Anticancer Properties of *Annona muricata*

**DOI:** 10.3390/molecules28155744

**Published:** 2023-07-29

**Authors:** Rehab H. Abdallah, Muneera S. M. Al-Saleem, Wael M. Abdel-Mageed, Al-Sayed R. Al-Attar, Youssef M. Shehata, Doaa M. Abdel-Fattah, Rahnaa M. Atta

**Affiliations:** 1 Department of Pharmacognosy, Faculty of Pharmacy, Zagazig University, Zagazig 44519, Egypt; rehabhamed2000@yahoo.com; 2 Department of Chemistry, Science College, Princess Nourah bint Abdulrahman University, P.O. Box 84428, Riyadh 11671, Saudi Arabia; 3 Department of Pharmacognosy, College of Pharmacy, King Saud University, P.O. Box 2457, Riyadh 11451, Saudi Arabia; 4 Department of Pharmacognosy, Faculty of Pharmacy, Assiut University, Assiut 71526, Egypt; 5 Department of Pathology, Faculty of Veterinary Medicine, Zagazig University, Zagazig 44519, Egypt; sayedattar50@gmail.com (A.-S.R.A.-A.); drdoaa30@yahoo.com (D.M.A.-F.); 6 Department of Biochemistry, Faculty of Veterinary Medicine, Zagazig University, Zagazig 44519, Egypt; rehabayman4117@gmail.com (Y.M.S.); rahnaaatta@yahoo.com (R.M.A.)

**Keywords:** *Annona muricata*, Annonaceae, UPLC-ESI-MS/MS, Bax, Bcl-2

## Abstract

*Annona muricate* is a tropical plant that is well-known for its edible fruit of therapeutic interest. LCMS/MS analyses were applied to identify phytoconstituents of the ethanolic extract of the whole fruits and the aqueous extract of the edible fruit part, in addition to the investigation of their anticancer properties against Ehrlich ascites carcinoma (EAC) in male albino mice. LCMS/MS analyses resulted in the identification of 388 components, representing a wide array of classes of compounds, including acetogenins as the major constituents, alkaloids, flavonoids, and phenolics. Among them, four compounds were tentatively characterized as new compounds (**1**–**4**), including an acid derivative, protocatechuic-coumaroyl-quinic acid (**1**), and three flavonoid derivatives, dihydromyricetin galloyl hexoside (**2**), apigenin gallate (**3**), and dihydromyricetin hexouronic acid hexoside (**4**). Induction with EAC cells resulted in abnormalities in the gene expression of pro-apoptotic genes (Bax and caspase-3) and anti-apoptotic gene (Bcl-2) in the tumor mass. Moreover, microscopic, histopathological, and immune-histochemical examinations of the tumor mass and liver tissues exhibited extensive growth of malignant Ehrlich carcinoma cells and marked hydropic degeneration of hepatocytes and infiltration by tumor cells to liver tissue with marked inflammatory reaction. These abnormalities were markedly ameliorated aftertreatment of EAC mice with *A. muricata* extracts.

## 1. Introduction

*Annona muricata* L., commonly known as soursop, Graviola, is a member of the family Annonaceae comprising approximately 130 genera and 2300 species [1]. It is a lowland tropical, fruit-bearing tree found in the rainforests of Africa, South America, and Southeast Asia, which has been shown to contain a wide range of constituents of interesting biological activities, such as antiviral, antiparasitic, anti-inflammatory, hypoglycemic, and anticancer activities [2,3]. The flesh and pulp fruits are rich in water, carbohydrate, vitamins, salts, and are ideal to be eaten readily or consumed as juice. *A. muricata* fruit and its juice are often used to treat fevers, increase milk in nursing mothers, and as a mordant for gastrointestinal orders such as diarrhea and dysentery [4]. *A. muricata* is reported to be a rich source of acetogenins, the major constituent of *Annona*, which has been identified to have cytotoxic activity against different types of cancer cells potentiated by the presence of flavonoids to enable maximum therapeutic effects [5,6].

Cancer is one of the deadliest diseases globally and especially in Western countries. According to the International Cancer Observatory, millions of people died in 2020 as a result of developing cancer [7]. This disorder results from genetic or epigenetic alterations in the somatic cells along with abnormal cell growth which may be spread to other body parts [8]. The cell death induced by an anticancer agent is programmed cell death or apoptosis. This is an important physiological process that is responsible for homeostatic mechanisms and the maintenance of cell populations in tissues [9]. The intervention of Bax, Bcl-2, and caspase-3 gene expression is an important factor for determining tumor susceptibility to a given anticancer agent [10]. Efforts are still being made to search for an impressive adjuvant anticancer therapy, from natural sources, that would lessen or even impede cancer progress.

The purpose of this study is to investigate the chemical constituents of both ethanolic and aqueous extracts of *A. muricata* using high-performance liquid chromatography (HPLC) linear ion trap mass spectrometry with negative and positive electrospray ionization modes and their anticancer effect on induced Ehrlich ascites carcinoma in adult albino mice.

## 2. Results and Discussion

### 2.1. Characterization of the Phytochemical Constituents

**Figure 1:**
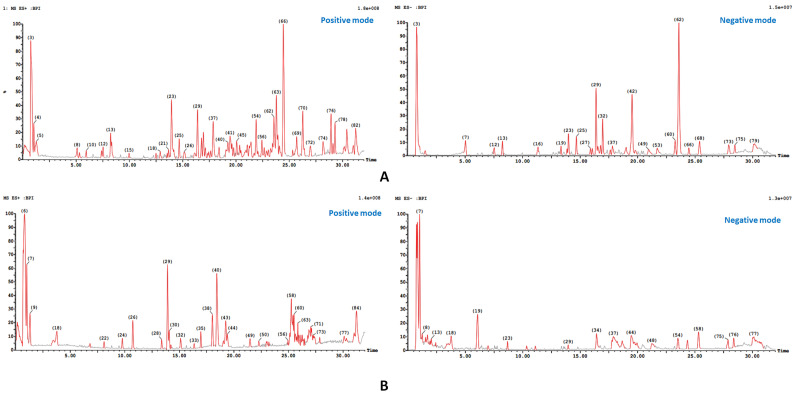
UPLC—ESI—MS/MS chromatogram of: (**A**) ethanolic extract of whole fruit, (**B**) water extract of the edible part of *A. muricata* in positive (+ve) and negative (−ve) ionization modes.

In the present work, qualitative LCMS/MS analyses of the phytochemical composition of the ethanolic extract of *A. muricata* whole fruits and the aqueous extract of the edible part of the fruit were carried out using HPLC–DAD–ESI-MS/MS in negative and positive ionization modes (Figure 1). The identification of the compounds based on the comparison of the results of ESI-MS/MS experiments expressed R_t_s and fragmentation patterns with those reported in studies collected from different databases, such as PubMed, Google Scholar, and Web of Science.

So far, 388 components have been tentatively identified from the ethanolic extract of *A. muricata* whole fruits and the water extract of the edible part of the fruit, as presented in Tables S1–S5. They represent a wide array of classes of compounds, mostly acetogenins (the major constituents of the genus *Annona*).

Acetogenins are polyketides, constituted of 35 or 37 carbon atoms arranged as a long alkyl chain terminated by a γ methyl γ lactone [11]. As represented in Table S1, 142 different types of acetogenins were tentatively characterized in this study. They included 36 acetogenins identified for the first time in *A. muricata* (Figure S1) and six identified for the first time from the genus *Annona* (Figure S2). The other 106 acetogenins were previously detected in this plant [1,6,11,12,13,14,15,16,17,18,19,20,21,22].

Alkaloids are the second major identified constituent, possessing isoquinoline-derived structures. In total, 37 alkaloids were identified, including 14 identified for the first time from *A. muricata* (Figure S3) and 11 identified for the first time from the genus *Annona* (Table S4) [23,24,25,26].

Interestingly, most of the identified compounds, including phenolics and flavonoids, were tentatively characterized for the first time from *A. muricata* and the genus *Annona* together with other miscellaneous phytochemicals (Tables S2, S3 and S5) [27,28,29,30,31,32,33,34,35,36,37,38,39,40,41,42,43,44,45,46,47,48,49,50,51,52,53,54,55,56,57,58,59,60,61,62,63,64,65,66,67,68,69,70,71,72,73,74,75,76,77,78,79,80,81,82].

**Figure 2:**
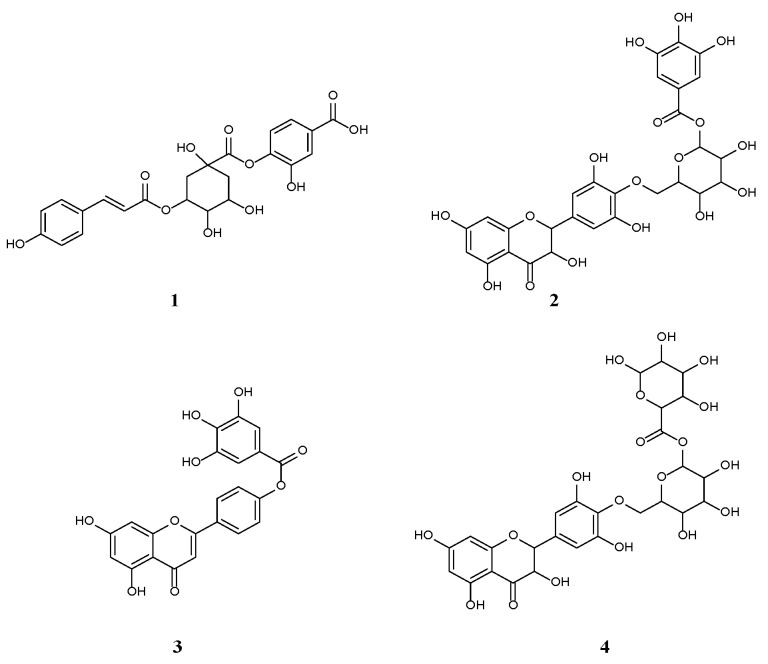
The structures of the new compounds (**1**–**4**) from *A. muricata* fruit.

Notably, four compounds were tentatively new and previously not described from nature. These included an acid derivative, protocatechuic-coumaroyl-quinic acid (**1**, Figure 2) (**146**, Table S2) and three flavonoid derivatives: dihydromyricetin galloyl hexoside (**2**, Figure 2) (**239**, Table S3), apigenin gallate (**3**, Figure 2) (**241**, Table S3), and dihydromyricetin hexouronic acid hexoside (**4**, Figure 2) (**244**, Table S3).

#### Structural Identification of New Compounds

**Figure 3:**
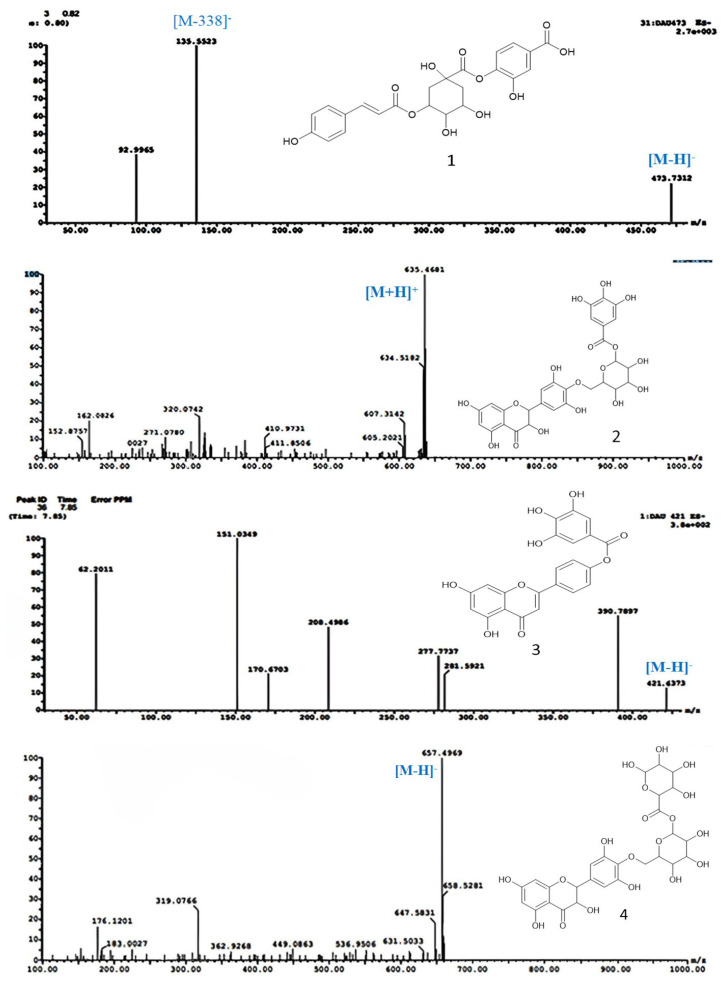
UPLC–ESI-MS/MS chromatograms of compounds (**1**–**4**) isolated from *A. muricata* extracts in positive (+) and negative (−) ionization modes.

Compound **1** (R_t_. 0.42 min) was tentatively assigned as protocatechuic coumaroyl quinic acid (Figure 2). The ESI–MS spectrum (Figure 3) showed a molecular ion peak at *m*/*z* 473 [M–H]^–^, and a base peak ion fragment at *m*/*z*135 [M–338]^–^, reflecting the existence of protocatechuic acid after the loss of coumaroyl quinic acid [30].

Compound **2** (R_t_. 10.92 min) was tentatively assigned as dihydromyricetin galloyl hexoside (Figure 2). The ESI–MS spectrum (Figure 3) showed a molecular ion peak at *m*/*z* 635 [M+H]^+^, and fragment ions at *m*/*z* 320 [M–galloyl hexoside]^+^, representing dihydromyricetin moiety 162 [M–dihydromyricetin galloyl moiety]^+^, and 152 [M–dihydromyricetin glucose]^+^. Thus, compound **2** was identified as dihydromyricetin galloyl hexoside [30].

Compound **3** (R_t_. 11.14 min) was tentatively assigned as apigenin gallate (Figure 2). The ESI-MS spectrum (Figure 3) showed a molecular ion peak at *m*/*z* 421 [M–H]^–^, fragment ions at *m*/*z* 170 [gallic acid]^+^, and a base peak at *m*/*z* 151 [M–H–270]^+^, corresponding to gallic acid after losing apigenin moiety. From this fragmentation pattern, compound **3** was tentatively identified as apigenin gallate [30].

Compound **4** (R_t_.12.36 min) was tentatively assigned as dihydromyricetin hexouronic acid hexoside (Figure 2). The ESI–MS spectrum (Figure 3) showed a molecular ion peak at *m*/*z* 657 [M–H]^–^, and fragment ions at *m*/*z* 176 and 162, resulting from the loss of hexouronic acid and hexoside moieties, respectively. From this fragmentation pattern, compound **4** was identified as dihydromyricetin hexouronic acid hexoside [30].

### 2.2. Molecular Findings

Most studies on the apoptotic activity of compounds were confirmed by Bax, Bcl-2, and caspase-3 regulation mechanisms. Notably, the significant loss or inactivation of caspase-3 impairs the induction of apoptosis, leading to a dramatic imbalance in growth dynamics and ultimately causing abnormal growth of human cancer cells [83].

**Figure 4:**
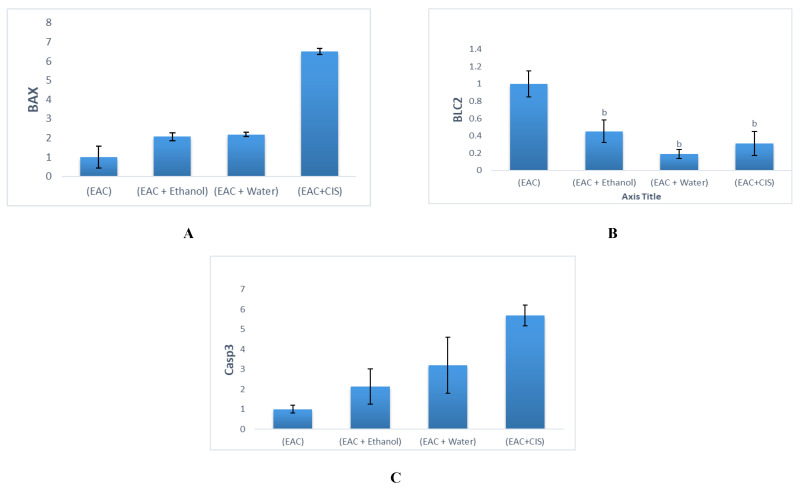
Analysis of PCR product of: (**A**) Bax gene, (**B**) Bcl-2 gene, and (**C**) Caspase-3 gene in tumor mass in different experimental groups by using RT-PCR. Results were expressed as mean ± SEM (*n* = 10). ^b^ *p <* 0.001 from the EAC group. *p <* 0.001 from the EAC + Ethanol group. *p <* 0.001 from EAC + Water groups.

This study revealed an elevation of the expression of the Bcl-2 gene with a concurrent reduction in the expression of Bax and caspase-3 genes upon cancer induction by Ehrlich tumor cells, as shown in Table 1 and Figure 4. Inversely, the expression of the Bcl-2 gene decreased, and the expression of Bax and caspase-3 genes increased after treatment with different *A. muricata* extracts, as shown in Table 1 and Figure 4. These results are in agreement with Awad et al. (2020) [84], who suggested that treatment with *A. muricata* extract triggered apoptosis by upregulating apoptotic genes Bax and caspase-3, and downregulating the anti-apoptotic Bcl-2 gene.

Moreover, our study agreed with the apoptotic effects found in vivo, where *A. muricata* inhibited the progression of orthotopically implanted breast and pancreatic tumors in mice, and chemically induced breast cancer in rats [85,86,87]. Interestingly, these in vivo studies showed different anticancer pathways. Furthermore, Torres et al., (2012)reported that Graviola can kill cancer cells through necrosis rather than apoptosis [86].

However, Dai et al., (2011), Syed Najmuddin et al., (2016), and Zeweil et al., (2019) reported mitochondrial-dependent apoptotic pathways, and they proved that Graviola is a powerful anticancer agent that does not cause additional harm to normal cells. This indicates the selectivity of Graviola on cancer cells and elicits the safety of Graviola on animals, unlike conventional anticancer drugs that show severe toxicity [85,87,88]. Moghadamtousi et al., (2015) also reported that Graviola induced apoptosis by activating caspases 3/7 and 9, upregulating Bax, and downregulating Bcl-2 at the mRNA and protein levels [1]. Therefore, Graviola upregulated Bax expression, resulting in the release of mitochondrial cytochrome *c* from mitochondria to the cytosol to form the apoptosome complex, which triggers activated caspase-3 expression [89].

### 2.3. Histopathological Findings

**Figure 5:**
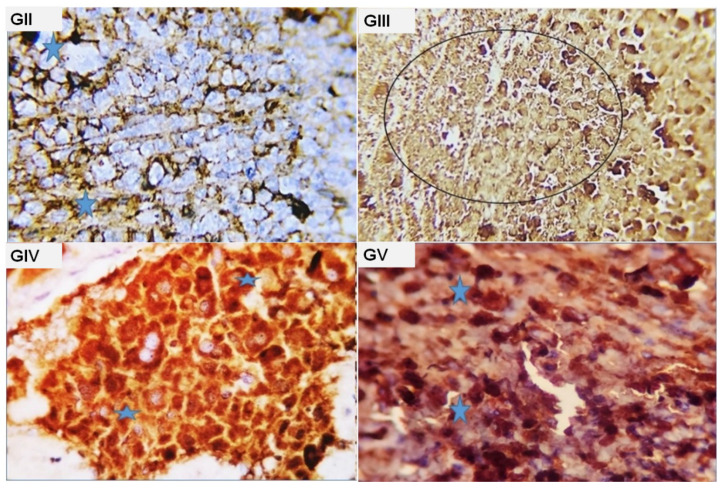
Photomicrograph from the tumor mass of different experimental groups immune-stained with P^53^. (**GII**): cells with negative staining reaction. A few apoptotic tumor cells appeared with dark positive cytoplasmic staining reaction (blue stars); (**GIII**): large numbers of necrotic cells with hazard staining reaction to the used marker. Some cells showed apoptotic reaction with dark positive brownish nuclear staining (dark circle); (**GIV**): marked apoptotic reaction in about 75–80% of the tumor cells as represented by dense brown cytoplasmic stainability of the affected tumor cells (blue stars); (**GV**): marked apoptotic reaction in about 75–80% of the tumor cells as represented by dense brown cytoplasmic stainability of the affected tumor cells (blue stars).

**Figure 6:**
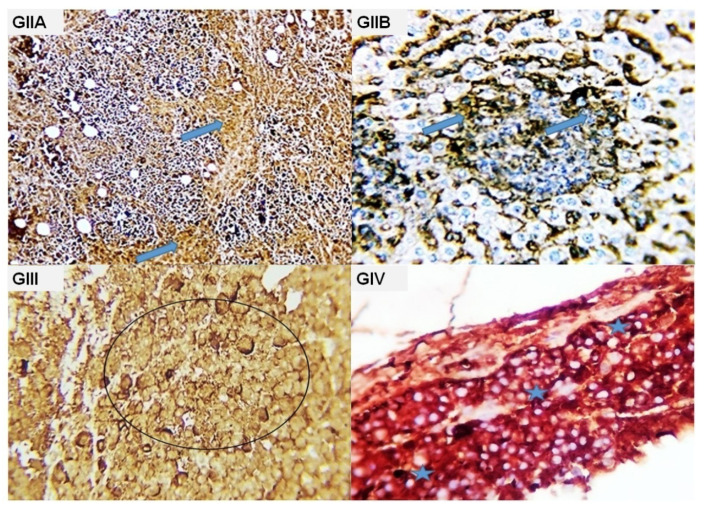
Photomicrograph from the tumor mass of different experimental groups immune-stained with CK. (**GIIA**, **GIIB**): Mark brownish cytoplasmic stainability of the malignant cells (blue arrows), (**GIII**): massive necrosis of the tumor cells with negative staining reaction. Some cells showed hazay cytoplasmic membrane brownish staining reaction (black circle); (**GIV**): remnants of tumor mass were seen with positive cytokeratin cytoplasmic staining reaction in most of the remaining cells (blue stars).

**Figure 7:**
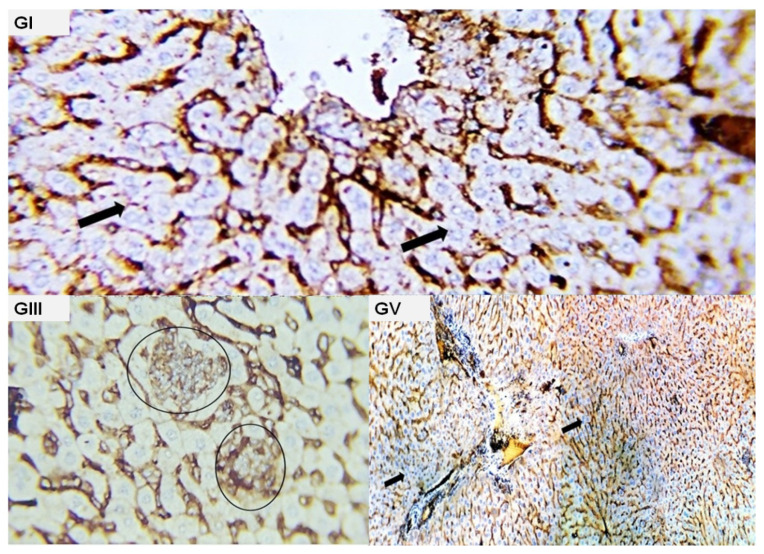
Photomicrograph from the liver of different experimental groups immune-stained with CK. (**GI**): normal hepatocytes free from any malignant cells (black arrow); (**GIII**): focal aggregates (deposit) of weak positive malignant cells (black circles); (**GV**): liver parenchyma appeared negative for any of metastatic cells, no positive reactivity for the used marker was detected (black arrow).

In the current study, histopathological evaluation of the plant extracts was conducted in a detailed manner. The therapeutic regimens were investigated according to histopathological, immune histochemical, and morphometric analyses. Histopathologically examined tumor mass of EAC and the possible hepatic metastatic cells, together with the associated immune cell response, degenerative, necrotic, and/or apoptotic changes in the different experimental groups (GII-GV), were recorded as shown in Figures S4 and S5 and Figure 5, Figure 6 and Figure 7.

In contrast with normal hepatic parenchyma with preserved portal triads (Figure S4, GIA and GIB), the greatest pathologic lesions were seen in mice that received EAC alone (GII). This was represented by large nodules in the peritoneal cavity and metastatic changes in the liver parenchyma, evidenced by atypical epithelial cells with large hyperchromatic nuclei with many mitotic activities (Figures S4 and S5, GII). Such findings were related to the high viability, mobilization, and metastatic probability of the used tumor cell line, and they were in accordance with the observation of Chakraborty et al., (2007) and Mansour et al., (2019),who reported similar histopathologic changes in mice that received EAC only [90,91].

Immunohistochemical and morphometric analyses of GII pointed out characteristic changes represented by the controversial reaction of the markers used, p^53^, and pancytokeratin (CK) (Figure 5 and Figure 6, GII; and Table 2). That is, the tumor cells were highly negative for p^53^ and highly positive for CK, indicating high viability and a high proliferative index of the tumor cell line. Such findings are in accordance with the result reported by Bassiony et al. (2014) [92].

Cisplatin treatment showed histopathological changes represented by complete tumoral necrotic changes with focal calcification of the intraperitoneal tumor mass, along with degenerative changes of hepatic cells, massive portal and interstitial inflammatory reaction, portal fibrosis, and multifocal hepatocellular coagulative necrosis (Figures S4 and S5, GIII). The aforementioned histopathological changes are drastic reactions attributed to the strong cytotoxic effect of cisplatin against tumor cells. Unfortunately, the cytotoxic effect is not limited to tumor cells, but it also includes normal healthy tissue, and it interferes with normal viable hepatic activities, so its therapeutic uses are limited [93]. These findings are in accordance with those reported by Do Amaral et al., (2008), Gong et al., (2015), and Niu et al. (2017) [94,95,96].

The immunohistochemical analysis of the group that received cisplatin (GIII) showed characteristic changes represented by large numbers of necrotic cells with a hazard staining reaction to the p^53^ marker and a negative staining reaction to the pan-cytokeratin marker with massive necrosis of the tumor cells (Figure 5 and Figure 6, GIII; and Table 2). Such findings are in accordance with those reported by Ikitimur-Armutak et al. (2015) [97].

Treatment with Graviola water extract from the edible part of the fruit (GIV) induced ameliorative changes and the disappearance of most pathological changes in tumor mass and metastatic tumor cells in the liver tissue. The tumor mass in the peritoneal cavity displayed necrotic and apoptotic changes in 80–85% of cells, while liver tissue sections showed normal hepatic parenchyma free of metastatic tumor cells and normal portal structure and blood vessels (Figures S4 and S5, GIV). Such changes could be attributed to the protective and ameliorative effects of the compounds in the edible part, with a promising feature to include the extracted purified active compounds to be used in combination with other chemical drugs to fight the highly social-facing battle of patients’ lives (tumor spread and death). The above-mentioned results are in agreement with Samin et al. (2016) and Alzergy et al. (2018) [98,99].

The group treated with ethanolic extract of the whole fruit (GV) showed a completely necrotic intraperitoneal tumor mass, and the hepatic parenchyma appeared normal and free of metastatic cells with the appearance of healthy active hepatocytes (Figures S4 and S5, GV). This was in accordance with findings reported by Abd El-Kaream et al. (2019) and Shukry et al. (2020) [100,101].

Moreover, the results for GIV and GV were confirmed by the results of the markers used, including p^53^ and cytokeratin (Figure 5, Figure 6 and Figure 7, GIV and GV; and Table 2). This revealed controversial signaling responses leading to marked apoptotic reactions in about 75–80% of tumor cells, represented by dense brown cytoplasmic stainability of the affected tumor cells. Such findings are in accordance with Prasad et al., (2019), who observed that acetogenins from *A. muricata* elevated the levels of p^53^ found in the nucleus upon treatment [102].

## 3. Materials and Methods

### 3.1. Plant Materials and Extract Preparation

Plant materials (fruits) were purchased from a local market in November 2020. The extraction of Graviola was conducted in the Faculty of Pharmacy, Zagazig University. The plant was identified and verified by Dr. Marwa Mohsen Eldemerdash, (Assistant Professor of Plant Taxonomy, Faculty of Science, Zagazig University). A voucher specimen (Ann.S-2) was deposited in the Herbarium of the Department of Pharmacognosy, Faculty of Pharmacy, Zagazig University, Egypt.

### 3.2. Extract Preparation

#### 3.2.1. Ethanolic Extraction of the Whole Fruits

Fresh fruits of *A. muricata* (5 kg) were cleaned, washed with distilled water, dried in the oven at 60 °C, milled into powder (1000 g), soaked in two liters of 95% ethanol for 48 h, and filtered through gauze and then filter paper. This step was repeated three times. The obtained alcoholic solution was evaporated and concentrated at room temperature by a rotary evaporator. The dried sticky extract (40 g) was dissolved using 1% Dimethyl sulfoxide DMSO in normal saline for pharmacological studies (each 1 gm of the sticky extract was dissolved in 1 mL of 1%DMSO) [103].

#### 3.2.2. Aqueous Extraction of the Fruit Pulps

The fruit pulp (the edible part inside the fruit) (4 kg) (without seeds) was extracted with distilled water at a percentage of 1:4 (pulp:distilled water). The mixture was filtered to discard any solid material. Finally, the filtrate extract was dried using the freeze dryer/lyophilizer machine to give the final product (700 mg), which was dissolved in 1 mL of 1%DMSO in normal saline for pharmacological studies [104].

### 3.3. UPLC-ESI-MS/MS Analyses of A. muricata Extracts

#### 3.3.1. LC/MS Instrument and Separation Technique

The sample solutions of the ethanolic extract of the whole fruits and the aqueous extract of the edible part of the fruit (100 μg/mL) was prepared using HPLC analytical-grade solvent of MeOH, filtered using a membrane disk filter (0.2 μm), and then subjected to LC-ESI-MS analysis. Samples of injection volumes (10 μL) were injected into the Ultra-performance liquid chromatography (UPLC) instrument (XEVO-TQD triple quadruple instrument, Waters Corporation, Milford, MA, USA) equipped with a reverse phase C-18 column (ACQUITY UPLC-BEH C_18_, 2.1 × 50 mm, 1.7 μm). The sample mobile phase was prepared by filtering using a 0.2 μm filter membrane disk and degassed by sonication before injection. Mobile phase elution was made with a flow rate of 0.2 mL/min using a gradient mobile phase comprising two eluents: eluent A was H_2_O acidified with 0.1% formic acid and eluent B was MeOH acidified with 0.1% formic acid. Elution was performed using the following gradient: 20% B, 0–1 min; 20–90% B, 1–18 min; and 20% B, 18–20 min. The parameters for analysis used negative and/or positive ion mode as follows: source temperature 150 °C, cone voltage 30 eV, capillary voltage 3 kV, desolvation temperature 450 °C, cone gas flow 50 L/h, and desolvation gas flow 900 L/h.

#### 3.3.2. Determination of UPLC-ESI-MS-MS

Mass spectra were detected in the ESI negative and/or positive ion modes between 50 *m*/*z* and 900 *m*/*z*. The peaks and spectra were processed using Maslynx 4.1 software and tentatively identified by comparing their retention time (R_t_) and mass spectrum with the reported data. For fragmentation collision energy, 40 eV was used.

### 3.4. Cytotoxic Activity

The cytotoxic study was conducted at the scientific and medical research center (ZSMRC) at Zagazig University. The experiment was conducted on 50 mice, which were randomly divided into 5 groups of 10 mice (Figure S6).

#### 3.4.1. Experimental Animals

Fifty male Swiss albino mice (25–30 g weight) were obtained from the animal house of the National Cancer Institute (NCI, Cairo University, Egypt). The animals were housed in metal cages and kept under standard laboratory conditions for aeration and room temperature at about 25 °C. They were provided with adequate rodent food and a water supply. Animals were handled and sacrificed ethically according to the procedures reviewed and approved by the Zagazig University research center’s institutional animal care and use committee (IACUC) under number *ZU-IACUC/2/F/91/2020*.

#### 3.4.2. Ehrlich Ascites Carcinoma

Ehrlich ascites carcinoma cells were gathered in vivo from male Swiss albino mice at the National Cancer Institute (NCI), Cairo University, Egypt.

#### 3.4.3. Cisplatin

Cisplatin was used as a positive control anticancer drug and obtained from Mylan Pharma, France, and freshly prepared before the treatment.

#### 3.4.4. Induction of Cancer by Ehrlich Ascites Carcinoma

The solid tumors were initiated by injecting (2.5 × 10^6^) cells subcutaneously in the right thigh of the lower limb of each mouse in GII-GV (*n* = 40). The tumors formed a week later [105].

#### 3.4.5. Treatment Regimen

Group I (the normal control group of healthy mice) received only the normal laboratory diet and tap water for 28 days. Group II (the positive control group) received only the EAC cells. Group III (the cisplatin-treated group) was treated with cisplatin at a dose of 2 mg/kg I.P. once weekly (day 10, day 17, and day 24) after 10 days of induction EAC for 28 days [106]. Group IV (the aqueous extract-treated group) received water extract at a daily dose of 200 mg/kg orally after 10 days of induction of EAC for 28 days [103]. Group V (ethanolic extract-treated group) received fruit extract at a daily dose of 200 mg/kg orally after 10 days of induction of EAC for 28 days [103].

#### 3.4.6. Tissue Samples

Immediately after collecting their blood, the animals were sacrificed and dissected. The tumor mass and liver were removed and washed with 0.9% NaCl to flush out any blood and blotted dry on paper. Parts of the tumor masses of all groups were kept at −80 °C until they were used to determine the gene expression of Bax, Bcl-2, and caspase-3 using reverse transcription polymerase chain reaction (RT-PCR). Other parts of the livers and tumor mass slices were kept in 10% neutral buffered formalin for histopathological and immunohistochemistry examinations.

#### 3.4.7. Molecular Determination

Total RNA was isolated from the tumor mass using an RNA extraction kit (Thermo Fisher Scientific, Inc.; Dreieich, Germany). Total RNA was reverse transcribed to complementary DNA (cDNA) using the HiSenScript^TM^ RH (-) cDNA Synthesis kit (iNtRON Biotechnology Co., Seongnam, Kyonggi-do, South Korea) in a Veriti 96-well thermal cycler (Applied Biosystems, Foster City, CA) for 60 min at 45 °C followed by 10 min at 85 °C. The primers sequence (5′-3′) used for PCR was as follows: Bax (forward primer: CTACAGGGTTTCATCCAG and reverse primer CCAGTTCATCTCCAATTCG); Bcl-2 (forward primer: GTGGATGACTGAGTACCT and reverse primer CCAGGAGAAATCAAACAGAG); caspase-3 (forward primer TGCGTGTGGAGTATTTGGATG and reverse primer TGGTACAGTCAGAGCCAACCTC); and GAPDH (forward primer GAGAAACCTGCCAAGTATG and reverse primer GGAGTTGCTGTTGAAGTC). Thermal cycling conditions were as follows: 1 cycle at 95 °C for 12 min for denaturation; 40 cycles at 95 °C for 15 s, 60 °C for 30 s for annealing, and 30 sec extensions at 72 °C. All samples were compared using the relative CT method.

#### 3.4.8. Histopathological Examination

Tissue (tumor mass and liver) from different groups of mice were quickly removed and fixed in 10% neutral buffered formalin for histopathological and immunohistochemical examination. Fixation comprised tissue immersion in 10% buffered formalin for 48 h, followed by removal of the fixative in distilled water for 30 min. Dehydration was then carried out by exposing the tissue to 70% alcohol for 120 min, followed by 90% alcohol for 90 min, and then two cycles of absolute alcohol, each for one hour. Then, the samples were cleaned in several changes of xylene. This consisted of tissue immersion for an hour in a mixture of 50% alcohol and 50% xylene, followed by pure xylene for one and a half hours. Samples were impregnated with molten paraffin wax and then embedded and blocked out. Paraffin sections (4–5 μm) were stained with hematoxylin and eosin [107]. Stained sections were examined for circulatory disturbances, inflammation, degeneration, apoptosis, necrosis, and any other pathological changes.

#### 3.4.9. Immunohistochemistry Investigation

The tissue sections were microwave treated. The presence of antigens in the tissues was identified by immunostaining, using a two-step process. The primary antibody was first bound to the related antigen, and then the reaction was visualized using a biotin–streptavidin (BSA) system [108]. 3,3′-Diaminobenzidine (DAB) was used for the permanent preparation, and hematoxylin was used for counterstaining. Five-micron-thick paraffin sections were mounted on positively charged glass slides (Biogenex, Freemont, CA, USA). Paraffin sections were soaked in xylene overnight and then passed through ethanol in concentrations of 100%, 95%, 75%, and 50%. The excess buffer was blotted off, and the slides were dried. One drop of supersensitive primary monoclonal antibodies (Pan-Cytokeratin and P^53^) was placed on the sections.

After incubation for 60 min, the slides were rinsed for 5 min in phosphate-buffered saline (PBS). Two drops of DAKO EnVision were applied for 20 min, followed by rinsing with PBS. DAB chromogen was applied for 10–20 min until the desired brown color was obtained, and then the slides were washed in the buffer to remove the DAB. Mayer’s hematoxylin (Hx) was used to counterstain the nuclei in the sections. In accordance with the degree of nuclear staining, sections were placed in Hx solution for 3–5 min, then washed in tap water and differentiated in acid–alcohol before being washed again in tap water. Air-dried slides were mounted with Canada balsam.

For myeloperoxidase immunohistochemistry, the heat was applied to the slides in a pressure cooker in Tris-buffered saline with 0.075% Tween-20 (pH 7.6) for 10 min to extract the antigen [109]. The samples were then incubated in 0.3% *v*/*v* H_2_O_2_ in methanol for 20 min at room temperature to inhibit endogenous peroxidase activity [110]. The sections were incubated at room temperature for 30 min with polyclonal rabbit antihuman myeloperoxidase antibody diluted 1:1500, and then stained. Immunostaining was performed using an avidin-biotin-horseradish peroxidase system (Vector Laboratories, Burlingame, CA) with 3-amino-9-ethyl carbazole as the chromogen for myeloperoxidase and diaminobenzidine for CD68 (Kirkegaard and Perry Laboratories, Gaithersburg, MD, USA).

#### 3.4.10. Morphometric Analysis

Image analysis slides were digitized using an Olympus digital camera (Olympus LC20-Tokyo, Japan) installed on an Olympus microscope (Olympus BX-50, Tokyo, Japan) with a 1/2× photo adaptor, using a 40× objective. The resulting images were analyzed on an Intel^®^ Core I3^®^-ased computer using Video Test Morphology 5.2 software (Mosco, Russia) with a specific built-in routine for immunohistostaining analysis and stain quantification. The system measured the area percentage of caspase-3 positive expression. Images from five slices per tissue were taken 200 μm apart. Five visions per slice were randomly chosen for assessing positive cells using image analysis software (JID801D). The average grayscale of the positive cells was calculated automatically [111].

### 3.5. Statistical Analysis

The results are expressed as mean values ± SEM (standard error of the mean). To assess the influence of the treatment groups on the different biochemical parameters, a one-way analysis of variance (ANOVA) was used. All analyses and charts were performed using the Statistical Package for Social Sciences version 28.0 (SPSS, IBM Corp., Armonk, NY, USA).

## 4. Conclusions

Our study represents the first comprehensive report on the phytochemical composition of the ethanolic extract of whole fruits and the aqueous extract of the edible part of the fruit of *A. muricata* cultivated in Egypt using HPLC–DAD–ESI-MS/MS and an investigation of their anticancer properties against EAC in male albino mice. The LCMS/MS analyses resulted in the tentative identification of 388 components that represented many types of classes of chemical compounds—including acetogenins, alkaloids, flavonoids, and phenolics—and mostly belonging to acetogenins. Four phenolic compounds were tentatively characterized for the first time in nature.

Biologically, induction of cancer with EAC cells resulted in a decrease in the gene expression of pro-apoptotic genes Bax and caspase-3 in tumor mass and a significant increase in anti-apoptotic gene Bcl-2 (EAC-treated group). Moreover, microscopic, histopathological, and immunohistochemical examination of the tumor mass and liver tissues of the EAC group exhibited extensive growth of malignant Ehrlich carcinoma cells, and the liver tissue showed marked hydropic degeneration of hepatocytes and infiltration by tumor cells, causing a marked inflammatory reaction. However, the administration of the different *A. muricata* extracts elevated the gene expression of pro-apoptotic Bax and caspase-3 and decreased the level of anti-apoptotic Bcl-2. Moreover, the microscopic, histopathological, and immunohistochemical abnormalities were markedly ameliorated after treating the EAC mice with *A. muricata* extracts, which appeared to be rich in biologically highly active cytotoxic constituents. This is what motivates us to recommend eating Graviola during chemotherapy to enhance its therapeutic benefits.

In conclusion, this represents the first chemical and biological study to explore these findings and is considered an addition to the bibliographic exploration of the chemical diversity and therapeutic values of *A. muricata* extracts. In the future, further comparative studies will be pursued to study the chemical constituents and the efficacy of these extracts in comparison with other commercial Annona products available in the market, which could aid in the development of new therapeutic agents and safe natural alternative therapies for the treatment of cancer.

## Figures and Tables

**Table 1:** The effect of cisplatin (2 mg/kg), *A. muricata* water extract (200 mg/kg), and *A. muricata* ethanol extract (200 mg/kg) on the pro-apoptotic and anti-apoptotic gene expression in tumor mass by using real-time PCR.

Parameters	GII (EAC)	GIII (EAC + Cisplatin)	GIV (EAC + Water)	GV (EAC + Ethanol)
BAX	1.00 ± 0.58	6.51 ± 0.71 ^b^	2.19 ± 0.11	2.07 ± 0.21
Bcl-2	1.00 ± 0.15	0.31 ± 0.09 ^b^	0.19 ± 0.05 ^b^	0.45 ± 0.13 ^b^
Casp-3	1.00 ± 0.20	5.71 ± 1.88 ^b^	3.21 ± 1.40	2.13 ± 0.88

Results were expressed as mean ± SEM (*n* = 10). from the control group. *p <* 0.01 and ^b^ *p* < 0.001 from the EAC group. *p <* 0.001 from the EAC + Ethanol group. *p <* 0.001 from EAC + Water groups.

**Table 2:** Morphometric analysis of P^53^ and CK.

Parameters	P^53^	CK
Groups		
Ethanol extract	2.44 ± 0.12 ^ab#^	2.78 ± 0.12 ^ab#^
Water extract	18.58 ± 0.48 *	16.40 ± 0.24 *
Cisplatin	19.35 ± 0.31 *	17.66 ± 0.41 *
Control −ve (GI)	2.24 ± 0.36	3.97 ± 0.69
Control +ve(GII)	19.05 ± 0.64 *	19.44 ± 0.61 *
*p* value	0.0001	0.0001

Results are expressed as mean ± SEM. * significantly different from group I (*p* < 0.001), ^a^ significantly different from group II (*p* < 0.01), ^b^ significantly different from cisplatin (*p* < 0.001), ^#^ significantly different from water extract (*p* < 0.001).

## Data Availability

The data presented in this work are available in the article.

## Supplementary Materials

The following supporting information can be downloaded at: https://www.mdpi.com/article/10.3390/molecules28155744/s1, Figure S1: Acetogenins identified for the first time from *A. muricata*; Figure S2: Acetogenins identified for the first time from genus *Annona*; Figure S3: Alkaloids identified for the first time from the genus *Annona*; Figure S4: Photomicrograph from the liver of different experimental groups (GII-GV); Figure S5: Photomicrograph of tumor mass of different experimental groups (GII-GV); Figure S6: Flow chart of animals’ cytotoxic study; Table S1: Acetogenins detected and characterized in *A. muricata* extracts; Table S2: Phenolics detected and characterized in *A. muricata* extracts; Table S3: Flavonoids and flavonoid derivatives detected and characterized in *A. muricata* extracts; Table S4: Alkaloids detected and characterized in *A. muricata* extracts; Table S5: Phytochemical compounds (miscellaneous) detected and characterized in *A. muricata* extracts.

## Sample Availability

Samples of the compounds are not available from the authors.

## References

[1] Moghadamtousi S.Z., Fadaeinasab M., Nikzad S., Mohan G., Ali H.M., Kadir H.A. (2015). *Annona muricata* (Annonaceae): A review of its traditional uses, isolated acetogenins and biological activities. Int. J. Mol. Sci..

[2] Hadisaputri Y.E., Habibah U., Abdullah F.F., Halimah E., Mutakin M., Megantara S., Abdulah R., Diantini A. (2021). Antiproliferation activity and apoptotic mechanism of soursop (*Annona muricata* L.) leaves extract and fractions on MCF7 breast cancer cells. Breast Cancer Targ. Ther..

[3] Mutakin M., Fauziati R., Fadhilah F.N., Zuhrotun A., Amalia R., Hadisaputri Y.E. (2022). Pharmacological activities of soursop (*Annona muricata* Lin.). Molecules.

[4] Asare G.A., Afriyie D., Ngala R.A., Abutiate H., Doku D., Mahmood S.A., Rahman H. (2015). Antiproliferative activity of aqueous leaf extract of *Annona muricata* L. on the prostate, BPH-1 cells, and some target genes. Integr. Cancer Ther..

[5] Dinda B., Dinda S., DasSharma S., Banik R., Chakraborty A., Dinda M. (2017). Therapeutic potentials of baicalin and its aglycone, baicalein against inflammatory disorders. Eur. J. Med. Chem..

[6] Avula B., Bae J.-Y., Majrashi T., Wu T.-Y., Wang Y.-H., Wang M., Ali Z., Wu Y.-C., Khan I.A. (2018). Targeted and non-targeted analysis of annonaceous alkaloids and acetogenins from *Asimina* and *Annona* species using UHPLC-QToF-MS. J. Pharm. Biom. Anal..

[7] Garcia-Oliveira P., Otero P., Pereira A.G., Chamorro F., Carpena M., Echave J., Fraga-Corral M., Simal-Gandara J., Prieto M.A. (2021). Status and challenges of plant-anticancer compounds in cancer treatment. Pharmaceuticals.

[8] Testa U., Petrucci E., Pasquini L., Castelli G., Pelosi E. (2018). Ovarian cancers: Genetic abnormalities, tumor heterogeneity and progression, clonal evolution and cancer stem cells. Medicines.

[9] Kim S.S., Cho H.J., Kang J.Y., Kang H.K., Yoo T.K. (2013). Inhibition of androgen receptor expression with small interfering RNA enhances cancer cell apoptosis by suppressing survival factors in androgen insensitive, late stage LNCaP cells. Sci. World J..

[10] Martinou J.-C., Youle R.J. (2011). Mitochondria in apoptosis: Bcl-2 family members and mitochondrial dynamics. Devel. Cell.

[11] Bermejo A., Figadère B., Zafra-Polo M.-C., Barrachina I., Estornell E., Cortes D. (2005). Acetogenins from Annonaceae: Recent progress in isolation, synthesis and mechanisms of action. Nat. Prod. Rep..

[12] Jacobo-Herrera N., Pérez-Plasencia C., Castro-Torres V.A., Martínez-Vázquez M., González-Esquinca A.R., Zentella-Dehesa A. (2019). Selective acetogenins and their potential as anticancer agents. Front. Pharm..

[13] Zafra-Polo M.C., González M.C., Estornell E., Sahpaz S., Cortes D. (1996). Acetogenins from Annonaceae, inhibitors of mitochondrial complex I. Phytochemistry.

[14] Champy P., Guérineau V., Laprévote O. (2009). MALDI-TOF MS profiling of annonaceous acetogenins in *Annona muricata* products for human consumption. Molecules.

[15] Gomes I.N.F., Silva-Oliveira R.J., Oliveira Silva V.A., Rosa M.N., Vital P.S., Barbosa M.C.S., Dos Santos F.V., Junqueira J.G.M., Severino V.G., Oliveira B.G. (2019). *Annona coriacea* Mart. fractions promote cell cycle arrest and inhibit autophagic flux in human cervical cancer cell lines. Molecules.

[16] Melot A., Fall D., Gleye C., Champy P. (2009). Apolar Annonaceous acetogenins from the fruit pulp of *Annona muricata*. Molecules.

[17] Adesanwo J.K., Akinloye A.A., Otemuyiwa I.O., Akinpelu D.A. (2020). Chemical Characteristics and Biological Activities of *Annona squamosa* Fruit Pod and Seed Extracts. J. Expl. Res. Pharm..

[18] Le Ven J., Schmitz-Afonso I., Lewin G., Brunelle A., Touboul D., Champy P. (2014). Identification of the environmental neurotoxins annonaceous acetogenins in an *Annona cherimolia* Mill. Alcoholic beverage using HPLC-ESI-LTQ-Orbitrap. J. Agri. Food Chem..

[19] Rupprecht J.K., Hui Y.-H., McLaughlin J.L. (1990). Annonaceous acetogenins: A review. J. Nat. Prod..

[20] Bonneau N., Baloul L., ba Ndob I.B., Senejoux F., Champy P. (2017). The fruit of *Annona squamosa* L. as a source of environmental neurotoxins: From quantification of squamocin to annotation of Annonaceous acetogenins by LC–MS/MS analysis. Food Chem..

[21] Ragasa C.Y., Galian R.F., Shen C.-C. (2014). Chemical constituents of *Annona muricata*. Der. Pharma Chem..

[22] Gu Z.-M., Zhou D., Wu J., Shi G., Zeng L., McLaughlin J.L. (1997). Screening for Annonaceous acetogenins in bioactive plant extracts by liquid chromatography/mass spectrometry. J. Nat. Prod..

[23] Gavamukulya Y., Maina E.N., Meroka A.M., Madivoli E.S., El-Shemy H.A., Magoma G., Wamunyokoli F. (2019). Liquid chromatography single quadrupole mass spectrometry (LC/SQ MS) analysis reveals presence of novel antineoplastic metabolites in ethanolic extracts of fruits and leaves of *Annona muricata*. Pharm. J..

[24] Rinaldi M.V., Díaz I.E., Suffredini I.B., Moreno P.R. (2017). Alkaloids and biological activity of beribá (*Annona hypoglauca*). Rev. Bras. Farm..

[25] Calixto N.O., Cordeiro M.S., Giorno T., Oliveira G.G., Lopes N.P., Fernandes P.D., Pinto A.C., Rezende C.M. (2017). Chemical constituents of *Psychotria nemorosa* Gardner and antinociceptive activity. J. Braz. Chem. Soc..

[26] Justino A.B., Florentino R.M., França A., Antonio Filho C., Franco R.R., Saraiva A.L., Fonseca M.C., Leite M.F., Espindola F.S. (2021). Alkaloid and acetogenin-rich fraction from *Annona crassiflora* fruit peel inhibits proliferation and migration of human liver cancer HepG2 cells. PLoS ONE.

[27] Rini Vijayan K., Raghu A. (2021). Polyphenolic profiling of two *Embelia* spp. endemic to South Western Ghats of India by liquid chromatography coupled with tandem mass spectrometry analysis. Nat. Prod. Res..

[28] Marzouk M.M., Elkhateeb A., Latif R.R.A., Abdel-Hameed E.-S.S., Kawashty S.A., Hussein S.R. (2019). *C*-glycosyl flavonoids-rich extract of *Dipcadi erythraeum* Webb & Berthel. bulbs: Phytochemical and anticancer evaluations. J. App. Pharm. Sci..

[29] Abu-Reidah I.M., Ali-Shtayeh M.S., Jamous R.M., Arráez-Román D., Segura-Carretero A. (2015). HPLC–DAD–ESI-MS/MS screening of bioactive components from *Rhus coriaria* L. (Sumac) fruits. Food Chem..

[30] Tan L., Jin Z., Ge Y., Nadeem H., Cheng Z., Azeem F., Zhan R. (2020). Comprehensive ESI-Q TRAP-MS/MS based characterization of metabolome of two mango (*Mangifera indica* L) cultivars from China. Sci. Rep..

[31] Mena P., Calani L., Dall’Asta C., Galaverna G., García-Viguera C., Bruni R., Crozier A., Del Rio D. (2012). Rapid and comprehensive evaluation of (poly) phenolic compounds in pomegranate (*Punica granatum* L.) juice by UHPLC-MSn. Molecules.

[32] Lachowicz S., Oszmiański J., Rapak A., Ochmian I. (2020). Profile and content of phenolic compounds in leaves, flowers, roots, and stalks of *Sanguisorba officinalis* L. determined with the LC-DAD-ESI-QTOF-MS/MS analysis and their in vitro antioxidant, antidiabetic, antiproliferative potency. Pharmaceuticals.

[33] El-Hawary S.S., Sobeh M., Badr W.K., Abdelfattah M.A., Ali Z.Y., El-Tantawy M.E., Rabeh M.A., Wink M. (2020). HPLC-PDA-MS/MS profiling of secondary metabolites from *Opuntia ficus-indica* cladode, peel and fruit pulp extracts and their antioxidant, neuroprotective effect in rats with aluminum chloride induced neurotoxicity. Saudi J. Biol. Sci..

[34] Mancini S., Nardo L., Gregori M., Ribeiro I., Mantegazza F., Delerue-Matos C., Masserini M., Grosso C. (2018). Functionalized liposomes and phytosomes loading *Annona muricata* L. aqueous extract: Potential nanoshuttles for brain-delivery of phenolic compounds. Phytomedicine.

[35] Plazonić A., Bucar F., Maleš Ž., Mornar A., Nigović B., Kujundžić N. (2009). Identification and quantification of flavonoids and phenolic acids in burr parsley (*Caucalis platycarpos* L.), using high-performance liquid chromatography with diode array detection and electrospray ionization mass spectrometry. Molecules.

[36] Kang J., Price W.E., Ashton J., Tapsell L.C., Johnson S. (2016). Identification and characterization of phenolic compounds in hydromethanolic extracts of *sorghum wholegrains* by LC-ESI-MSn. Food Chem..

[37] Zhang Y., Shi P., Qu H., Cheng Y. (2007). Characterization of phenolic compounds in *Erigeron breviscapus* by liquid chromatography coupled to electrospray ionization mass spectrometry. Rapid Commun. Mass Spectrom. Int. J. Devoted Rapid Dissem. Up Minute Res. Mass Spectrom..

[38] El-Sayed M.A., Al-Gendy A.A., Hamdan D.I., El-Shazly A.M. (2017). Phytoconstituents, LC-ESI-MS profile, antioxidant and antimicrobial activities of *Citrus x limon* L. Burm. f. cultivar variegated pink lemon. J. Pharm. Sci. Res..

[39] Saftić L., Peršurić Ž., Fornal E., Pavlešić T., Pavelić S.K. (2019). Targeted and untargeted LC-MS polyphenolic profiling and chemometric analysis of propolis from different regions of Croatia. J. Pharm. Biomed. Anal..

[40] Boukhalkhal S., Gourine N., Pinto D.C., Silva A.M., Yousfi M. (2020). UHPLC-DAD-ESI-MSn profiling variability of the phenolic constituents of *Artemisia campestris* L. populations growing in Algeria. Biocata. Agr. Biotech..

[41] Oliveira-Alves S.C., Vendramini-Costa D.B., Cazarin C.B.B., Júnior M.R.M., Ferreira J.P.B., Silva A.B., Prado M.A., Bronze M.R. (2017). Characterization of phenolic compounds in chia (*Salvia hispanica* L.) seeds, fiber flour and oil. Food Chem..

[42] Yisimayili Z., Abdulla R., Tian Q., Wang Y., Chen M., Sun Z., Li Z., Liu F., Aisa H.A., Huang C. (2019). A comprehensive study of pomegranate flowers polyphenols and metabolites in rat biological samples by high-performance liquid chromatography quadrupole time-of-flight mass spectrometry. J. Chrom. A.

[43] Jiménez-González A., Quispe C., Bórquez J., Sepúlveda B., Riveros F., Areche C., Nagles E., García-Beltrán O., Simirgiotis M.J. (2018). UHPLC-ESI-ORBITRAP-MS analysis of the native Mapuche medicinal plant palo negro (*Leptocarpha rivularis* DC.–Asteraceae) and evaluation of its antioxidant and cholinesterase inhibitory properties. J. Enz. Inh. Med. Chem..

[44] Lantzouraki D.Z., Sinanoglou V.J., Tsiaka T., Proestos C., Zoumpoulakis P. (2015). Total phenolic content, antioxidant capacity and phytochemical profiling of grape and pomegranate wines. RSC Adv..

[45] Coria-Téllez A.V., Obledo-Vázquez E.N., Padilla-Camberos E., González-Ávila M., Martínez-Velázquez M. (2019). Bioactivity, nutritional property, and rapid chemical characterization of aqueous extract of *Annona muricata* leaf from Mexico. Trop. J. Pharm. Res..

[46] Larrazábal-Fuentes M.J., Fernández-Galleguillos C., Palma-Ramírez J., Romero-Parra J., Sepúlveda K., Galetovic A., González J., Paredes A., Bórquez J., Simirgiotis M.J. (2020). Chemical profiling, antioxidant, anticholinesterase, and antiprotozoal potentials of *Artemisia copa* Phil. (Asteraceae). Front. Pharmacol..

[47] Cristofori V., Caruso D., Latini G., Dell’Agli M., Cammilli C., Rugini E., Bignami C., Muleo R. (2011). Fruit quality of Italian pomegranate (*Punica granatum* L.) autochthonous varieties. Eur. Food Res. Tech..

[48] Souza D.O., dos Santos Sales V., de Souza Rodrigues C.K., de Oliveira L.R., Lemos I.C.S., de Araújo Delmondes G., Monteiro Á.B., do Nascimento E.P. (2018). Phytochemical analysis and central effects of *Annona muricata* Linnaeus: Possible involvement of the gabaergic and monoaminergic systems. Iran. J. Pharm. Res..

[49] Simirgiotis M.J., Quispe C., Mocan A., Villatoro J.M., Areche C., Bórquez J., Sepúlveda B., Echiburu-Chau C. (2017). UHPLC high resolution orbitrap metabolomic fingerprinting of the unique species *Ophryosporus triangularis* Meyen from the Atacama Desert, Northern Chile. Rev. Bras. Farm..

[50] Izquierdo-Vega J.A., Arteaga-Badillo D.A., Sánchez-Gutiérrez M., Morales-González J.A., Vargas-Mendoza N., Gómez-Aldapa C.A., Castro-Rosas J., Delgado-Olivares L., Madrigal-Bujaidar E., Madrigal-Santillán E. (2020). Organic acids from Roselle (*Hibiscus sabdariffa* L.)—A brief review of its pharmacological effects. Biomedicines.

[51] George V.C., Kumar D.N., Suresh P., Kumar R.A. (2015). Antioxidant, DNA protective efficacy and HPLC analysis of *Annona muricata* (soursop) extracts. J. Food Sci. Tech..

[52] Zhang Y., Liu X., Gao S., Qian K., Liu Q., Yin X. (2018). Research on the neuro-protective compounds in *Terminalia chebula* Retz extracts in-vivo by UPLC–QTOF-MS. Act. Chrom..

[53] Beelders T., De Beer D., Stander M.A., Joubert E. (2014). Comprehensive phenolic profiling of *Cyclopia genistoides* (L.) Vent. by LC-DAD-MS and-MS/MS reveals novel xanthone and benzophenone constituents. Molecules.

[54] Bystrom L.M., Lewis B.A., Brown D.L., Rodriguez E., Obendorf R.L. (2008). Characterisation of phenolics by LC–UV/Vis, LC–MS/MS and sugars by GC in *Melicoccus bijugatus* Jacq.‘Montgomery’fruits. Food Chem..

[55] Kammerer D., Carle R., Schieber A. (2004). Characterization of phenolic acids in black carrots (*Daucus carota* ssp. sativus var. atrorubens Alef.) by high-performance liquid chromatography/electrospray ionization mass spectrometry. Rapid Commun. Mass Spectr..

[56] Falcão S.I., Vale N., Gomes P., Domingues M.R., Freire C., Cardoso S.M., Vilas-Boas M. (2013). Phenolic profiling of Portuguese propolis by LC–MS spectrometry: Uncommon propolis rich in flavonoid glycosides. Phytoch. Anal..

[57] Nuncio-Jáuregui N., Nowicka P., Munera-Picazo S., Hernández F., Carbonell-Barrachina Á.A., Wojdyło A. (2015). Identification and quantification of major derivatives of ellagic acid and antioxidant properties of thinning and ripe Spanish pomegranates. J. Func. Foods.

[58] Ibrahim T.A. (2012). Chemical composition and biological activity of extracts from *Salvia bicolor* Desf. growing in Egypt. Molecules.

[59] Benayad Z., Gómez-Cordovés C., Es-Safi N.E. (2014). Characterization of flavonoid glycosides from fenugreek (*Trigonella foenum-graecum*) crude seeds by HPLC–DAD–ESI/MS analysis. Inter J. Mol. Sci..

[60] Gu D., Yang Y., Abdulla R., Aisa H.A. (2012). Characterization and identification of chemical compositions in the extract of *Artemisia rupestris* L. by liquid chromatography coupled to quadrupole time-of-flight tandem mass spectrometry. Rap. Comm. Mass. Spect..

[61] Saldanha L.L., Vilegas W., Dokkedal A.L. (2013). Characterization of flavonoids and phenolic acids in *Myrcia bella* cambess. Using FIA-ESI-IT-MSn and HPLC-PAD-ESI-IT-MS combined with NMR. Molecules.

[62] Abdel-Hameed E.-S.S., Bazaid S.A., Salman M.S. (2013). Characterization of the phytochemical constituents of Taif rose and its antioxidant and anticancer activities. BioMed Res. Int..

[63] Šuković D., Knežević B., Gašić U., Sredojević M., Ćirić I., Todić S., Mutić J., Tešić Ž. (2020). Phenolic profiles of leaves, grapes and wine of grapevine variety vranac (*Vitis vinifera* L.) from Montenegro. Foods.

[64] Ambigaipalan P., de Camargo A.C., Shahidi F. (2016). Phenolic compounds of pomegranate byproducts (outer skin, mesocarp, divider membrane) and their antioxidant activities. J. Agric. Food Chem..

[65] Al-Yousef H.M., Hassan W.H., Abdelaziz S., Amina M., Adel R., El-Sayed M.A. (2020). UPLC-ESI-MS/MS profile and antioxidant, cytotoxic, antidiabetic, and antiobesity activities of the aqueous extracts of three different *Hibiscus* Species. J. Chem..

[66] Flamini R. (2013). Recent applications of mass spectrometry in the study of grape and wine polyphenols. Int. Sch. Res. Not..

[67] Hassan W.H., Abdelaziz S., Al Yousef H.M. (2019). Chemical composition and biological activities of the aqueous fraction of *Parkinsonea aculeata* L. growing in Saudi Arabia. Arab. J. Chem..

[68] Zhao H.-Y., Fan M.-X., Wu X., Wang H.-J., Yang J., Si N., Bian B.-L. (2013). Chemical profiling of the Chinese herb formula Xiao-Cheng-Qi decoction using liquid chromatography coupled with electrospray ionization mass spectrometry. J. Chrom. Sci..

[69] Ozarowski M., Piasecka A., Paszel-Jaworska A., Chaves D.S.d.A., Romaniuk A., Rybczynska M., Gryszczynska A., Sawikowska A., Kachlicki P., Mikolajczak P.L. (2018). Comparison of bioactive compounds content in leaf extracts of *Passiflora* incarnata, *P. caerulea* and *P. alata* and in vitro cytotoxic potential on leukemia cell lines. Rev. Bras. Farm..

[70] Ben Said R., Hamed A.I., Mahalel U.A., Al-Ayed A.S., Kowalczyk M., Moldoch J., Oleszek W., Stochmal A. (2017). Tentative characterization of polyphenolic compounds in the male flowers of *Phoenix dactylifera* by liquid chromatography coupled with mass spectrometry and DFT. Int. J. Mol. Sci..

[71] Li S., Lin Z., Jiang H., Tong L., Wang H., Chen S. (2016). Rapid identification and assignation of the active ingredients in fufang banbianlian injection using HPLC-DAD-ESI-IT-TOF-MS. J. Chrom. Sci..

[72] Lee S.-H., Kim H.-W., Lee M.-K., Kim Y.J., Asamenew G., Cha Y.-S., Kim J.-B. (2018). Phenolic profiling and quantitative determination of common sage (*Salvia plebeia* R. Br.) by UPLC-DAD-QTOF/MS. Eur. Food Res. Tech..

[73] Abdelaziz S., Hassan W.H., Elhassanny A.E., Al-Yousef H.M., Elsayed M.A., Adel R. (2020). Ultra performance liquid chromatography-tandem mass spectrometeric analysis of ethyl acetate fraction from saudi *Lavandula coronopifolia* Poir and evaluation of its cytotoxic and antioxidant activities. J. Herbmed Pharm..

[74] Taamalli A., Arráez-Román D., Abaza L., Iswaldi I., Fernández-Gutiérrez A., Zarrouk M., Segura-Carretero A. (2015). LC-MS-based metabolite profiling of methanolic extracts from the medicinal and aromatic species *Mentha pulegium* and *Origanum majorana*. Phytoch. Anal..

[75] Wang Y., Yang L., He Y.Q., Wang C.H., Welbeck E.W., Bligh S.A., Wang Z.T. (2008). Characterization of fifty-one flavonoids in a Chinese herbal prescription Longdan Xiegan Decoction by high-performance liquid chromatography coupled to electrospray ionization tandem mass spectrometry and photodiode array detection. Rapid Commun. Mass Spect. Int. J. Devoted Rapid Dissem. Up Minute Res. Mass Spectrom..

[76] Bielecka M., Pencakowski B., Stafiniak M., Jakubowski K., Rahimmalek M., Gharibi S., Matkowski A., Ślusarczyk S. (2021). Metabolomics and DNA-Based Authentication of Two Traditional Asian Medicinal and Aromatic Species of *Salvia* subg. Perovskia. Cells.

[77] Uysal S., Zengin G., Sinan K.I., Ak G., Ceylan R., Mahomoodally M.F., Uysal A., Sadeer N.B., Jekő J., Cziáky Z. (2021). Chemical characterization, cytotoxic, antioxidant, antimicrobial, and enzyme inhibitory effects of different extracts from one sage (*Salvia ceratophylla* L.) from Turkey: Open a new window on industrial purposes. RSC Adv..

[78] Haq F.U., Ali A., Akhtar N., Aziz N., Khan M.N., Ahmad M., Musharraf S.G. (2020). A high-throughput method for dereplication and assessment of metabolite distribution in *Salvia* species using LC-MS/MS. J. Adv. Res..

[79] Friščić M., Bucar F., Hazler Pilepić K. (2016). LC-PDA-ESI-MSn analysis of phenolic and iridoid compounds from *Globularia* spp. J. Mass Spec..

[80] Zhu Z., Zhang H., Zhao L., Dong X., Li X., Chai Y., Zhang G. (2007). Rapid separation and identification of phenolic and diterpenoid constituents from Radix *Salvia miltiorrhizae* by high-performance liquid chromatography diode-array detection, electrospray ionization time-of-flight mass spectrometry and electrospray ionization quadrupole ion trap mass spectrometry. Rapid Commun. Mass Spect. Int. J. Devoted Rapid Dissem. Up Minute Res. Mass Spectrom..

[81] Hou Z.-F., Xie Z.-X., Tu Y.-Q., Li Y. (2002). Triterpenes and triterpene glycosides from *Salvia tricupis*. Indian J. Chem. B.

[82] Jia C., Zhu Y., Zhang J., Yang J., Xu C., Mao D. (2017). Identification of glycoside compounds from tobacco by high performance liquid chromatography/electrospray ionization linear ion-trap tandem mass spectrometry coupled with electrospray ionization orbitrap mass spectrometry. J. Braz. Chem. Soc..

[83] Park S.-H., Kim M., Lee S., Jung W., Kim B. (2021). Therapeutic potential of natural products in treatment of cervical cancer: A review. Nutrients.

[84] Awad M.G., Ali R.A., Abd El-Monem D.D., El-Magd M.A. (2020). Graviola leaves extract enhances the anticancer effect of cisplatin on various cancer cell lines. Mol. Cell. Toxicol..

[85] Dai Y., Hogan S., Schmelz E.M., Ju Y.H., Canning C., Zhou K. (2011). Selective growth inhibition of human breast cancer cells by graviola fruit extract in vitro and in vivo involving downregulation of EGFR expression. Nutr. Cancer.

[86] Torres M.P., Rachagani S., Purohit V., Pandey P., Joshi S., Moore E.D., Johansson S.L., Singh P.K., Ganti A.K., Batra S.K. (2012). Graviola: A novel promising natural-derived drug that inhibits tumorigenicity and metastasis of pancreatic cancer cells in vitro and in vivo through altering cell metabolism. Cancer Lett..

[87] Zeweil M.M., Sadek K.M., Taha N.M., El-Sayed Y., Menshawy S. (2019). Graviola attenuates DMBA-induced breast cancer possibly through augmenting apoptosis and antioxidant pathway and downregulating estrogen receptors. Environ. Sci. Poll. Res..

[88] Syed Najmuddin S.U.F., Romli M.F., Hamid M., Alitheen N.B., Nik Abd Rahman N.M.A. (2016). Anti-cancer effect of *Annona Muricata* Linn Leaves Crude Extract (AMCE) on breast cancer cell line. BMC Complement. Altern. Med..

[89] Moghadamtousi S.Z., Kadir H.A., Paydar M., Rouhollahi E., Karimian H. (2014). *Annona muricata* leaves induced apoptosis in A549 cells through mitochondrial-mediated pathway and involvement of NF-κB. BMC Complement. Altern. Med..

[90] Chakraborty T., Bhuniya D., Chatterjee M., Rahaman M., Singha D., Chatterjee B.N., Datta S., Rana A., Samanta K., Srivastawa S. (2007). *Acanthus ilicifolius* plant extract prevents DNA alterations in a transplantable Ehrlich ascites carcinoma-bearing murine model. World J. Gastr..

[91] Mansour M.A., Salama A.F., Ibrahim W.M., Shalaan E.S. (2019). Assessment of Autophagy as Possible Mechanism of the Antitumor Effects of Arsenic Trioxide and/or Cisplatin on Ehrlich Ascites Carcinoma Model. Alex. J. Vet. Sci..

[92] Bassiony H., Sabet S., Salah El-Din T.A., Mohamed M.M., El-Ghor A.A. (2014). Magnetite nanoparticles inhibit tumor growth and upregulate the expression of P53/P16 in Ehrlich solid carcinoma bearing mice. PLoS ONE.

[93] Zhang Q., Lu Q.-B. (2021). New combination chemotherapy of cisplatin with an electron-donating compound for treatment of multiple cancers. Sci. Rep..

[94] Do Amaral C.L., Francescato H.D.C., Coimbra T.M., Costa R.S., Darin J.D.a.C., Antunes L.M.G., Bianchi M.D.L.P. (2008). Resveratrol attenuates cisplatin-induced nephrotoxicity in rats. Arch. Toxicol..

[95] Gong C., Qian L., Yang H., Ji L.-l., Wei H., Zhou W.-b., Qi C., Wang C.-h. (2015). Hepatotoxicity and pharmacokinetics of cisplatin in combination therapy with a traditional Chinese medicine compound of Zengmian Yiliu granules in ICR mice and SKOV-3-bearing nude mice. BMC Complement. Altern. Med..

[96] Niu C., Ma M., Han X., Wang Z., Li H. (2017). Hyperin protects against cisplatin-induced liver injury in mice. Acta Cir. Bras..

[97] Ikitimur-Armutak E.I., Sonmez K., Akgun-Dar K., Sennazli G., Kapucu A., Yigit F., Yilmaz V.T., Ulukaya E. (2015). Anticancer effect of a novel palladium–saccharinate complex of terpyridine by inducing apoptosis on Ehrlich ascites carcinoma (EAC) in Balb-C mice. Anticaner Res..

[98] Alzergy A., Haman M.R., Shushni M.A., Almagtouf F.A. (2018). Phyto-pharmaceuticals and biological study on graviola (*Annona muricata* L.) fruit and dietary supplement of graviola sold on the Libyan market as a cancer cure against TCA induce hepatotoxicity in mice. Cancer Biol.Ther..

[99] Samin B., Fachrial E., Refilda, Chaidir Z., Almahdy A. (2016). Protective Effect of Aqueous Extract of *Annona muricata* Leaves Against Copper Induced Hepatotoxicity in Experimental Rats. Res. J. Pharm. Biol. Chem. Sci..

[100] Shukry M., El-Shehawi A.M., El-Kholy W.M., Elsisy R.A., Hamoda H.S., Tohamy H.G., Abumandour M.M., Farrag F.A. (2020). Ameliorative effect of graviola (*Annona muricata*) on mono sodium glutamate-induced hepatic injury in rats: Antioxidant, apoptotic, anti-inflammatory, lipogenesis markers, and histopathological studies. Animals.

[101] Abd El-Kaream S.A. (2019). Biochemical and biophysical study of chemopreventive and chemotherapeutic anti-tumor potential of some Egyptian plant extracts. Biochem. Bioph. Rep..

[102] Prasad S.K., Varsha V., Devananda D. (2019). Anti-cancer properties of *Annona muricata* (L.): A Review. Medicinal Plants–Int. J. Phytomed. Relat. Ind..

[103] De Sousa O.V., Vieira G.D.-V., De Pinho J.d.J.R., Yamamoto C.H., Alves M.S. (2010). Antinociceptive and anti-inflammatory activities of the ethanol extract of *Annona muricata* L. leaves in animal models. Int. J. Mol. Sci..

[104] Syahida M., Maskat M., Suri R., Mamot S., Hadijah H. (2012). Soursop (*Annona muricata* L.): Blood hematology and serum biochemistry of sprague-dawley rats. Intern. Food Res. J..

[105] Abd Eldaim M.A., Tousson E., Soliman M.M., El Sayed I.E.T., Abdel Aleem A.A.H., Elsharkawy H.N. (2021). Grape seed extract ameliorated Ehrlich solid tumor-induced hepatic tissue and DNA damage with reduction of PCNA and P53 protein expression in mice. Environ. Sci. Pollut. Res. Int..

[106] El-Naggar S.A. (2011). Lack of the beneficial effects of mirazid (*Commiphora molmol*) when administered with chemotherapeutic agents on Ehrlich ascetic carcinoma bearing mice. Biol. Res..

[107] Suvarna S., Layton C., Bancroft J. (2013). The Hematoxylins and Eosin. Bancroft’s Theory and Practice of Histological Techniques.

[108] Hsu S.-M., Raine L., Fanger H. (1981). A comparative study of the peroxidase-antiperoxidase method and an avidin-biotin complex method for studying polypeptide hormones with radioimmunoassay antibodies. Am. J. Clin. Pathol..

[109] Carson H.J., Reddy V., Taxy J.B. (1998). Proliferation markers and prognosis in Merkel cell carcinoma. J. Cutan. Pathol..

[110] Harlow E., Lane D. (1988). A Laboratory Manual.

[111] Hashish H., Kamal R. (2015). Effect of curcumin on the expression of Caspase-3 and Bcl-2 in the spleen of diabetic rats. J. Exp. Clin. Anat..

