# Supplementary material for: LCMS/MS Phytochemical Profiling, Molecular, Pathological, and Immune-Histochemical Studies on the Anticancer Properties of Annona muricata
Source: Molecules. 2023 Jul 29;28(15):5744. doi: 10.3390/molecules28155744 (PMC10421100; doi:10.3390/molecules28155744)
Supplement: Supplementary file 1 [file molecules-28-05744-s001.zip › molecules-2492167-supplementary.pdf]

LCMS/MS Phytochemical profiling, molecular, pathological and immune-histochemical studies on the anticancer properties of *Annona muricata*

Rehab H. Abdallah, Muneera S. M. Al-Saleem, Wael M. Abdel-Mageed, Al-sayed R. Al-attar, Youssef M. Shehata, Doaa M. Abdel-Fattah, Rahnaa M. Atta

Content

No.	Title	Pages
**Table S1**	Acetogenins detected and characterized in *A. muricata* extracts	3-7
**Fig. S1**	Acetogenins identified for the first time from *A. muricata*.	8,9
**Fig. S2**	Acetogenins identified for the first time from genus Annona	10
**Table S2**	Phenolics detected and characterized in *A. muricata* extracts	11-13
**Table S3**	Flavonoids and flavonoid derivatives detected and characterized in *A. muricata* extracts	14-16
**Table S4**	Alkaloids detected and characterized in *A. muricata* extracts	16-17
**Fig. S3**	Alkaloids identified for the first time from the genus Annona	18
**Table S5**	Phytochemical compounds (miscellaneous) detected and characterized in *A. muricata* extracts	19-20
**Fig. S4**	Photomicrograph from the liver of different experimental groups (GII-GV).	21
**Fig. S5**	Photomicrograph of tumor mass of different experimental groups (GII-GV).	22
**Fig. S6**	Flow chart of animals' cytotoxic study	23

Table S1. Acetogenins detected and characterized in *A. muricata* in both ethanolic extract of fruit & water extract of the edible part of the fruit by using HPLC–DAD/QTOF-MS in positive and negative ionization modes.

No	Cpd-name	Rt	Mwt	M^+	M^-	Ms/Ms Fragment	Ref.	E	W
1	✿Muridienin-1	16.76	514	-	513	514	[11]	✓	×
2	✿✿Annofolin	16.87	253	-	252	252	[12]	✓	✓
3	✿Muricatacin	17.98	284	-	283	283	[1]	✓	✓
4	✿Muricatin-C	18.06	610	-	609	609,577	[11,13]	✓	✓
5	✿Muricin-J	19.05	414	415	-	415	[1]	✓	✓
6	✿✿Squamocin-O1/O2	19.15	638	639	-	621,603	[6]	✓	×
7	✿✿✿Trilobalicin	19.62	610	-	609	609,557,539,521	[6]	✓	✓
8	✿Norcorydine	19.64	327	328	-	328,192	[1,6]	✓	✓
9	✿Epomuricenin A,B	19.70	530	-	529	529,292,152	[1,11]	✓	✓
10	✿Epomusenin A	19.84	558	559	-	558,336,299,265	[1,11]	✓	✓
11	✿Muricatenol	19.92	608	-	607	607	[1,11]	✓	✓
12	✿Annomuricin A	19.99	612	-	611	611,593,575,539	[1,11]	×	✓
13	✿Annomuricin B	20.06	612	-	611	611,593,575,539	[1,11]	×	✓
14	✿✿Squamostatin A	20.07	638	639	-	621,603	[6]	✓	×
15	✿✿Uvaricin	20.08	648	649	-	649,613,595	[11]	✓	×
16	✿Gigantecin	20.30	638	639	-	621,603,533,403,391	[6,14]	✓	×
17	✿Annonacin	20.46	596	597	-	579,561,543,525	[1,6]	✓	✓
18	✿✿✿Parvifloracin	20.48	610	-	609	609,557,539,521	[6]	✓	✓
19	✿Annomuricin C	20.53	612	-	611	611,593,575,539	[1,11]	×	✓
20	✿Muricatocin A	20.68	612	-	611	611,593,575,539	[1,11]	×	✓
21	✿Annonacin-10-one	20.69	594	-	593	577,559,541,523	[1,6]	✓	✓
22	✿✿Purpurediolin	20.82	638	639	639	349,531	[6]	✓	×
23	✿Muricatocin B	20.84	612	-	611	611,593,575,539	[1,11]	×	✓
24	✿Muricatocin C	20.85	612	-	611	611,593,575,539	[1,11]	×	✓
25	✿✿✿Salzmanolin	20.87	654	655	-	655,619	[6]	✓	×
26	✿Iso-annonacin-10-one	20.88	594	-	593	577,559,541,523	[1,6]	✓	✓

27	✿✿Annoglaucin	20.91	638	639	-	621,567,549,531	[6,15]	✓	×
28	✿*Cis*-annonacin-10-one	20.91	594	-	593	577,559,541,523	[1,6]	✓	✓
29	✿Muridienin-2	20.95	542	-	541	542	[11]	✓	×
30	✿Muricin K	21.05	442	-	441	441	[1]	×	✓
31	✿✿10-hydroxyasmicin	21.07	638	639	-	621,567,549,531	[6]	✓	×
32	✿Muricadienin	21.39	514	-	513	514	[1,11]	✓	×
33	✿Muricatalacin	21.40	612	-	611	611,593,575,539	[11]	×	✓
34	✿Monticristin	21.41	574	-	-	575,557	[1,11]	✓	×
35	✿Cohebin C	21.50	576	-	-	577,558	[1,11]	✓	✓
36	✿✿Montanacin B/C	21.71	610	-	609	609,557,539,521	[11]	✓	✓
37	✿Cohebin D	21.73	576	577	-	577,558	[1,11]	✓	✓
38	✿Annomuricin E	21.75	612	-	611	611,593,575,539	[11]	×	✓
39	✿Muricapentocin	21.86	612	-	611	611,593,575,539	[11]	×	✓
40	✿2,4-*trans*-isoannonacin-10-one	21.90	594	-	593	577,559,541,523	[1,6]	✓	✓
41	✿Epumurinin B	21.95	532	533	-	531,295,277,237	[16]	✓	✓
42	✿Annonacin-A	22.05	596	597	-	579,561,543,525	[1,6]	✓	✓
43	✿✿Bullatanocin	22.06	638	639	-	621,603,567,549	[6,17]	✓	×
44	✿✿ 12,15-*cis*-bullatanocin	22.08	638	639	-	621,603,567,549	[6,17]	✓	×
45	✿10-Hydroxytrilobacin	22.09	638	639	-	621,603,567	[6]	✓	×
46	✿Dieporeticanin	22.40	574	575	-	575,557	[11,13]	✓	×
47	✿✿Squamocin B	22.45	594	-	593	577,559,541,523	[11,18]	✓	✓
48	✿✿Montacin(cis)	22.62	610	-	609	609,557,539,521	[11]	✓	✓
49	✿✿Asimitrin	22.65	638	639	-	621,603,567	[6]	✓	×
50	✿✿4-Hydroxy trilobin	22.85	638	639	-	621,603,567	[6]	✓	×
51	✿✿ 4-Acetyl-annonacin	22.87	638	639	-	621,603,567	[6]	✓	×
52	✿✿4-Acetylxylomaticin	22.90	638	639	-	621,603,567	([6]	✓	×
53	✿✿Bullatalicin	23.00	638	639	-	527,469,329,309,241	[6,18,19]	✓	×
54	✿✿Bullatetrocin	23.01	638	639	-	621,603,567	[6]	✓	×
55	✿*Cis*-annonacin	23.03	596	597	-	579,561,543,525	[1,6]	✓	✓
56	✿✿Coreaheptocin A	23.16	642	-	641	641,607	[15]	✓	×

57	●*Cis*-goniothalamacin	23.17	596	597	-	484,384,308	[1,11]	✓	✓
58	●Corepoxylone	23.19	560	561	-	651	[1,13]	✓	✓
59	●*Cis*-corossolone	23.22	578	579	-	579,445,338,298	[1,18]	✓	×
60	●Javoricin	23.25	596	597	-	597,579,661	[1,11]	✓	✓
61	●Muridienin-3	23.43	542	-	541	542	[1,11]	✓	×
62	●Muridienin-4	23.72	542	-	541	542	[1,11]	✓	×
63	●Annonacinone(cis)	23.79	594	-	593	577,559,541,523	[6,20]	✓	✓
64	●●Annoheptocin B	23.90	672	-	671	671	[15]	✓	×
65	●Annocinone	23.93	594	-	593	577,559,541,523	[1]	✓	✓
66	●Arianacin	23.95	596	597	-	597,579,661	[1,11],	✓	✓
67	●●Coreaheptocin B	24.07	642	-	641	641,607	[15]	✓	×
68	●Annoreticuin-9-one	24.08	594	-	593	577,559,541,523	[1,21]	✓	✓
69	●*Cis*-annoreticuin	24.09	596	597	-	597,579,661	[1,21]	✓	✓
70	●AnocatacinAand B	24.10	578	579	-	579,561,543,525,507	[1,11]	✓	×
71	●●Neoannonin (squamocin J)	24.26	578	579	-	579,310,270,243	[13]	✓	×
72	●Gigantetrocin A	24.28	596	597	-	597,579,661	[1,11]	✓	✓
73	●Motrilin	24.34	622	623	-	510,320,390,392	[6,18]	✓	✓
74	●Epomusenin B	24.95	558	559	-	558,336,299,265	[1,11].	✓	✓
75	●Annomutacin	25.02	624	-	623	623,607,589,553	[1,6]	✓	✓
76	●Gigantetrocin B	25.11	596	597	-	597,579,661	[1,11].	✓	✓
77	●●Annoheptocin A	25.15	670	671	-	671	[15]	✓	×
78	●Muricatetrocin A/B	25.23	596	597	-	597,579,661	[1,11]	✓	✓
79	●●Goniotriocin	25.27	636	637	-	637,619	[15]	✓	✓
80	●Muricin A	25.37	596	597	-	597,579,661	[1,11]	✓	✓
81	●● 9-oxo-asimicinone	25.48	636	637	-	637,619	[14]	✓	✓
82	●Muricatin A	25.63	612	-	611	611,593,575,539	[11,13]	×	✓
83	●Muricin B	25.70	596	597	-	597,579,661	[1,11]	✓	✓
84	●Muricatin B	25.75	612	-	611	611,593,575,539	[11,13]	×	✓
85	●Chatenaytrienin 1+2	25.76	540	-	539	539	[1,11]	×	✓
86	●Muricatalin	25.86	612	-	611	611,593,575,539	[11]	×	✓
87	●Annopentocin A+B	25.94	612	-	611	611,593,575,539	[1,11]	×	✓
88	●Muricin L	25.95	442	-	441	441	[1]	×	✓
89	●Muricin-N	25.96	414	415	-	415	[1]	✓	✓

90	●Epoxymurin A,B	25.97	530	-	529	529,292,152	[1,11]	✓	✓
91	●Sabadelin	26.13	530	-	529	529,292,152	[1,11]	✓	✓
92	●Murihexol	26.19	614	615	-	614,578	[1,11]	×	✓
93	●Donhexocin	26.22	614	615	-	614,578	[1]	×	✓
94	●●Squamocin I/K	26.23	578	579	-	579,561,543,507	[13,22]	✓	×
95	●Anohexocin	26.26	628	629	-	629,611,593,575	[1,11]	✓	✓
96	●Squamocin	26.27	622	623	-	510,320,390,392	[18]	✓	✓
97	●Murihexocin A	26.38	628	629	-	629,611,593,575	[1,11]	✓	✓
98	●Murihexocin B	26.46	628	629	-	629,611,593,575	[1,11]	✓	✓
99	●Cis-uvariamicin I	26.48	592	593	-	593,755	[1,11]	✓	✓
100	●Cis-uvariamicin IV	26.61	592	593	-	593,755	[1,11]	✓	✓
101	●Muricatenol	26.62	608	609	-	609,590,554	[1,11].	✓	✓
102	●Cis-uvariamicin II	26.64	592	593	-	593,755	[1,11]	✓	✓
103	●16,19-cis murisolin	26.72	580	581	-	581,527,509	[1,11].	✓	×
104	●Murihexocin C	26.70	628	629	-	629,611,593,575	[1,11]	✓	✓
105	●●●Asimilobin	26.76	578	579	-	579,561,543,507	[6]	✓	×
106	●Muricin-H	26.79	580	581	-	581,527,509	[1,11].	✓	×
107	●Muricin C	26.86	596	597	-	597,579,661	[1,11].	✓	✓
108	●Muricin F	26.97	594	-	593	577,559,541,523	[1,11]	✓	✓
109	●Muricin G	27.17	594	-	593	577,559,541,523	[1,20]	✓	✓
110	●Annomuricatin A	27.26	558	559	-	559	[1]	✓	✓
111	●Muricatatin	27.35	614	615	-	614,578	[11]	×	✓
112	●Annomuricatin C	27.35	558	559	-	559	[1]	✓	✓
113	●Reticulatacin	27.36	592	593	-	593,755	[1,11]	✓	✓
114	●Corrosolin	27.73	580	581	-	468,336	[1,11].	✓	×
115	●Chatenaytrienin-1	27.79	512	-	511	511,457	[1,11].	✓	✓
116	●●● Isomurisolenin	27.80	578	579	-	579,561,543,507	[6]	✓	×
117	●Muricoreacin	27.87	628	629	-	629,611,593,575	[1,11]	✓	✓
118	●●Annonisin	28.17	610	-	609	498,426,324	[18]	✓	✓
119	●Cis-annomontacin	28.59	624	-	623	623,587	[1,6]	✓	✓
120	●Bullatalacin	28.61	622	623	-	510,336,408	[18]	✓	✓
121	●Xylomaticin	28.87	624	-	623	623	[1]	✓	✓
122	●2,4(cis/trans)-10-R-annocin-A-one	28.93	596	597	-	597,579,661	[1,11].	✓	✓
123	●Annomontacin	28.97	624	-	623	623,605,589	[1]	✓	✓
124	●Montancin-A	28.99	640	-	639	639,567,550	[6,14]	✓	×
125	●Chatenaytrienin-2	29.09	512	-	511	511,457	[1]	✓	✓
126	●Iso-annonacin	29.29	596	597	-	597,579,661	[1,19]	✓	✓
127	●Asmicin	29.33	622	623	-	510,320,392,390	15,18]	✓	✓

128	●Gigantetronenin	29.50	622	623	-	623,,605,,587,551	[1]	✓	✓
129	●Annocatalin	29.60	596	597	-	597,579,661	[1].	✓	✓
130	●●●Asitrilobin A/C/D	30.01	624	-	623	623,587	[6]	✓	✓
131	●2,4 cis-isoannonacin	30.04	596	597	-	597,579,661	[1]	✓	✓
132	●Epumurinin A	30.05	532	533	-	531,295,277,237	[16]	✓	✓
133	●●Rollidecin- C	30.07	578	579	-	579,409,380,309,241	[22]	✓	×
134	●Annopentocin C	30.10	612	-	611	611,593,575,539	[1,11]	×	✓
135	● Muricin M	30.11	422	-	441	441	[1]	×	✓
136	●Annomuricinone D	30.13	612	-	611	611,593,575,539	[11]	×	✓
137	●longifolicin	30.18	580	581	-	527,509	[1,11].	✓	×
138	●●Annoglaxin	30.40	610	-	609	498,426,324	[11]	✓	✓
139	●2,4 trans-isoannonacin	30.67	596	597	-	597,579,661	[1]	✓	✓
140	●2,4 cis-gigantetrocinone	30.83	596	597	-	597,579,661	[1]	✓	✓
141	●●Annomontanin C	31.01	610	-	609	498,426,324	[11]	✓	✓
142	●2,4 trans-gigantetrocinone	31.25	596	597	-	597,579,661	[1]	✓	✓

●Compounds previously identified in *Annona muricata*
●●Compounds identified for the first time in *Annona muricata*
●●●Compounds identified for the first time in *Annona genus*

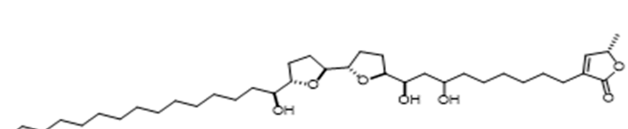

2 Annofolin

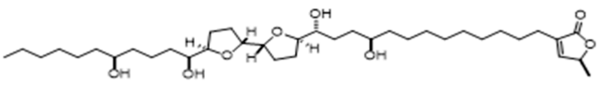

6 Squamocin-O_1

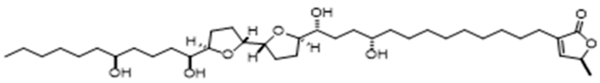

6 Squamocin-O_2

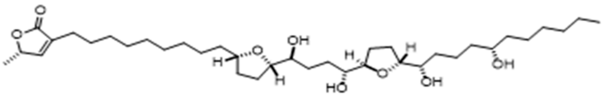

14 Squamostatin A

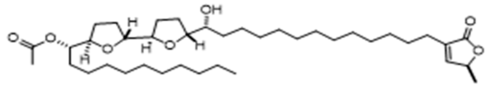

15 Uvaricin

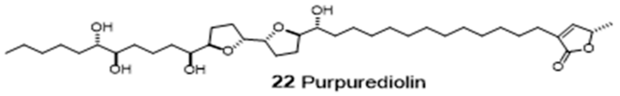

22 Purpurediolin

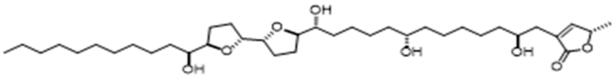

27 Annoglaucin

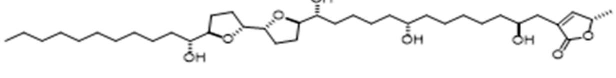

31 10-hydroxyasmicin

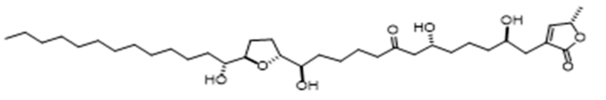

36 Montanacin B

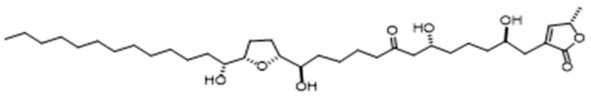

36 Montanacin C

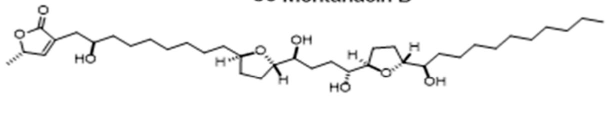

43 Bullatanocin

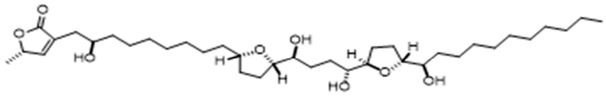

44 12,15-cis-bullatanocin

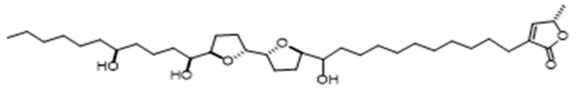

47 squamocin B

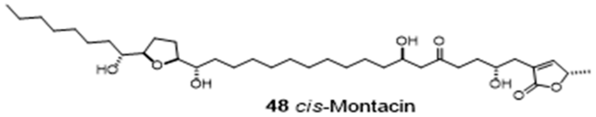

48 *cis*-Montacin

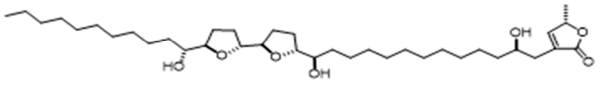

49 Asimitrin

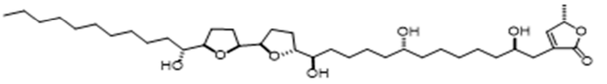

50 4-Hydroxytrilobin

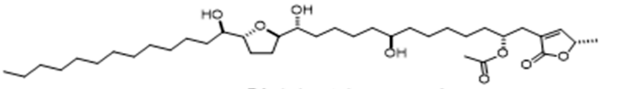

51 4-Acetyl-annonacin

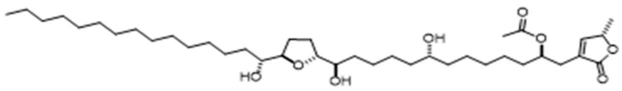

52 4-Acetylxylomaticin

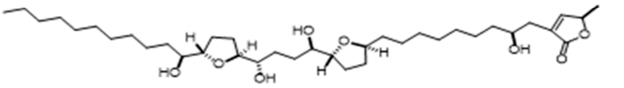

53 Bullatalicin

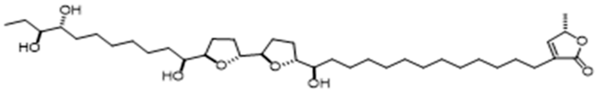

54 Bullatetrocin

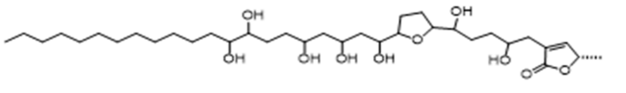

56 Coreaheptocin A
67 Coreaheptocin B

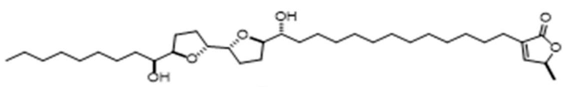

71 squamocin J

Figure S1. Acetogenins identified for the first time from *Annona muricata*.

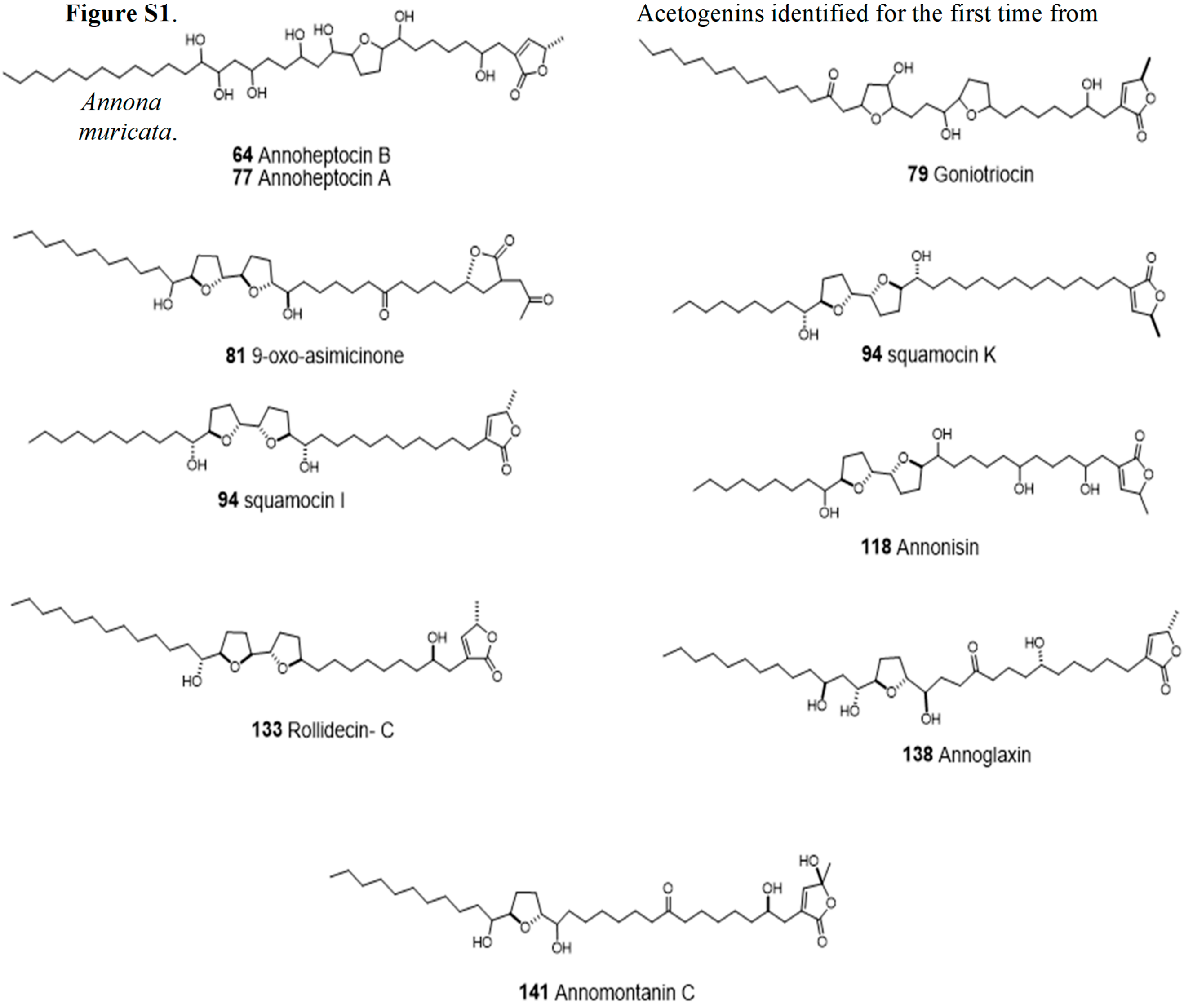

Figure S1. Cont. Acetogenins identified for the first time from *Annona muricata*.

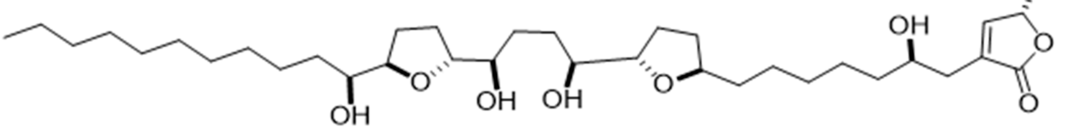

7 Trilobalicin

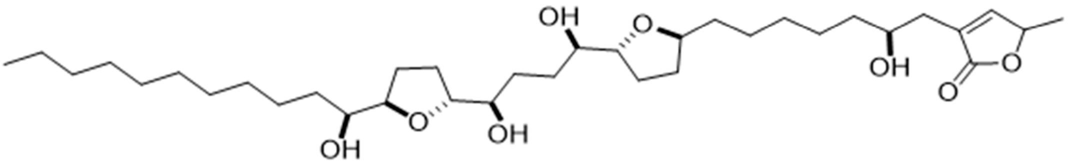

18 Parvifloracin

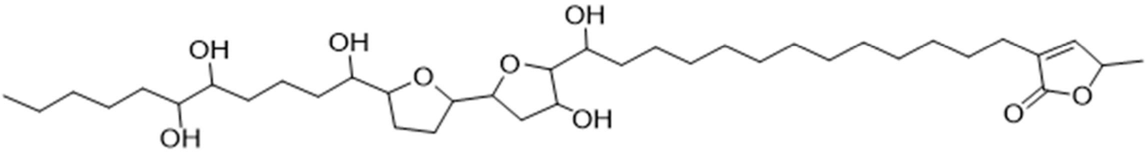

25 Salzmanolin

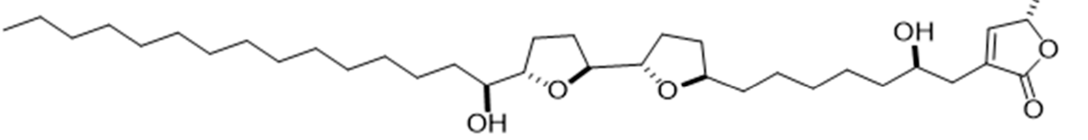

105 Asimilobin

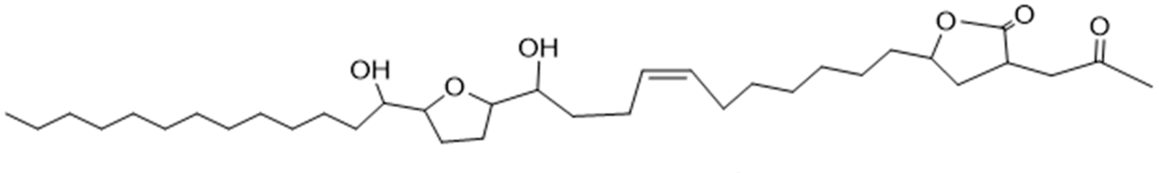

116 Isomurisolenin

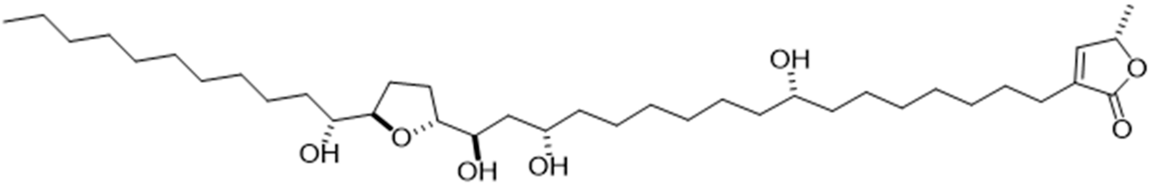

130 Asitriloin D

Figure S2. Acetogenins identified for the first time from genus *Annona*.

Table S2. Phenolics detected and characterized in *A. muricata* in both ethanolic extract of fruit & water extract of the edible part of the fruit by using HPLC–DAD/QTOF-MS in positive and negative ionization modes.

NO	Cpd-name	RT	Mwt	M^+	M^-	Ms/Ms Fragment	Ref.	E	W
143	••Methyl catechol	0.15	140	141	-	141,140,113,109	[27]	×	✓
144	••Malonylcoumaroylquinic acid	0.27	422	-	421	421,146	[28]	✓	✓
145	••Succinyl-dicaffeoylquinic acid	0.41	616	617	-	617,517,103	[29]	✓	✓
146	✓ Protochatechuic-coumaroylquinic acid	0.42	474	-	473	473,338,135	(New Compound) [30]	×	✓
147	••Trigalloyl-glucose	0.47	650	651	-	605,479,301,299	[31]	✓	✓
148	••Digalloyl hexose malic acid	0.70	600	601	-	601,303,297	[29]	✓	×
149	••Disuccinyl-caffeoylquinic acid	0.71	554	555	-	555,537 ,353,191	[32]	✓	×
150	•Dicaffeoylquinic acid	0.72	516	-	515	515,354	[1]	✓	×
151	•Feuloulcaffeoylquinic acid	1.53	530	-	529	529, 180	[1]	✓	✓
152	••Diferuloyl-syringic acid	1.70	550	551	-	550,354,197	[33]	✓	×
153	••Galloyl-valonic acid biloactone	0.80	662	-	661	469,393,169	[29]	✓	✓
154	•3-*O*-caffeic-quinic acid+ procyandrv.	0.82	578	-	577	577,289,,191,179	[34]	✓	×
155	••Coumaroylshikimic acid pentoside	1.13	452	453	-	453,321,132	[30]	✓	✓
156	••Di-caffeic acid	1.14	342	-	341	341,160,179,280	[35]	✓	✓
157	••Caffeoylshikimic acid	1.15	336	337	-	336,174,162	[36]	✓	×
158	••Malonyl-mono CQA3	1.29	440	441	-	352,265,173,87	[37]	✓	✓
159	•Caffeoylquinic acid (chlorogenic acid)	4.00	354	355	-	355,193	[1,34]	✓	✓
160	••Abscisic acid-*O*-glu-HMG	1.30	586	587	-	587,482	[38]	×	✓
161	•• Phenyl acetic acid pentoside	1.32	268	269	-	269,136,133	[39]	✓	✓
162	• Ferulic acid hexoside	1.33	356	-	355	356,193,162	[1]	✓	✓
163	••Coumaric acid hexosidedrv.	1.47	422	-	421	421,164	[40]	✓	✓
164	•• Caffeic acid hexose	1.52	342	-	341	341,179	[27]	✓	✓
165	••Ellagic acid-rhamnoside	1.70	448	449	-	302,228	[31]	✓	×
166	•• Caffeic acid arabinose	1.71	312	313	-	312,179	[41]	✓	✓
167	••Ellagic acid-hexose	1.72	464	465	-	301,300	[42]	×	✓
168	••Galloyl HHDP-gluconate	1.74	650	651	-	497,301,257,229	[31]	✓	✓
169	••Protocatechuic acid glucoside	2.01	316	-	315	315,153	[43]	✓	✓
170	•• Ferulic acid arabinose	2.03	326	-	325	193,177,149,134	[41]	✓	✓
171	•Coumaric acid glucoside	2.04	326	-	325	325,249,165	[1]	✓	✓
172	••Coumaric acid-rhamnose	2.05	310	-	309	309,146	[27]	✓	✓

173	●● Syringic acid pentoside	2.07	330	331	-	331,198,133	[30]	✓	✓
174	●● Dimer of tergallagic acid-hexose	2.08	632	633	-	451,301,299	[44]	✓	×
175	● Caffeic acid	2.09	180	-	179	179	[45]	✓	✓
176	● Quinic acid	2.28	192	193	-	193,173,129,113	[46]	✓	✓
177	● Gallic acid	2.46	170	-	169	169,152	[1,45]	×	✓
178	● Citric acid	2.54	192	193	-	104,85	[47]	✓	✓
179	●Ellagic acid	2.69	302	303	-	285,275,229	[48]	✓	✓
180	●●Shikimic acid hexoside	**2.75**	336	337	-	336,174,162	[36]	✓	×
181	●●Shikimic acid	2.76	174	175	-	175,174(100%),131	[28]	✓	✓
182	●●Sinapic acid drv.	2.80	436	437	-	437,224	[30]	✓	✓
183	●● Caffeoyl-shikimic acid drv.	2.84	586	587	-	387,336	[49]	×	✓
184	●●Caftaric acid	2.90	312	313	-	313,179	[30]	✓	✓
185	●● Hibiscus acid	3.05	190	191	-	191	[50]	✓	×
186	●*p*-coumaric acid	3.06	164	165	-	164,146	[17],[51]	✓	✓
187	●*p*-coumaric acid-methyl ether	**3.09**	178	179	-	179,165	[1]	✓	✓
188	●●Dicaffeic acid drv.	3.18	616	617	-	617,,341	[27]	✓	✓
189	●●Hydroxyl ferulic acid drv.	3.25	318	319	-	319,210	[30]	✓	✓
190	●● Ferulic acid drv.	3.27	273	-	272	272,158,132,125	[27]	✓	✓
191	●●*p*-coumaric acid drv.	3.43	360	361	-	361,214,147,118	[39])	✓	✓
192	●● Gallic acid drv.	3.70	266	-	265	265,170	[30]	✓	✓
193	●●Coumaric acid drv.	3.82	294	-	293	293,163	[30]	✓	✓
194	●●Ellagic acid drv.	3.92	440	441	-	441,302	[30]	✓	✓
195	●● Gallic acid drv.	3.93	398	399	-	399,171	[30]	×	✓
196	●●Galloflavin	4.06	278	279	-	279,197	[52]	✓	×
197	●●Trigalloyllevoglucosan	4.07	618	619	-	619,153,109	[29]	✓	✓
198	●● Hibiscus acid drv.	4.44	308	309	-	309,172	[30]	✓	×
199	●● Di-*O*-galloyl-HHDD protoquercitol I	5.09	618	619	-	301	[29]	✓	✓
200	●● Quinic acid drv.	5.49	330	331	-	331,193,175	[30]	✓	✓
201	●● Maclurin-3-O-glucoside	5.77	424	425	-	353,341,329,287,261	[53]	✓	×
202	●● Malic acid hexose drv.	5.80	436	437	-	437,348,297	[29]	✓	✓
203	●● Caffeic acid drv.	6.70	378	-	377	377,179(100%)	[54]	✓	✓

204	●●Maclurindrv.	6.99	360	361	-	361,262	[53]	✓	✓
205	●●Muclurin-drv.	7.03	380	381	-	381,262	[53]	✓	✓
206	●● Gallic-malic acid-drv.	7.07	398	399	-	399,286	[29]	×	✓
207	●●Hibiscus acid drv.	7.09	452	453	-	453,190	[30]	✓	✓
208	●● Ferulic acid drv.	7.29	382	383	-	383,206,149,134	[55]	✓	✓
209	●Eugenol	7.49	164	165	-	165	[17]	✓	✓
210	●● Hydroxyl citric acid drv.	8.43	336	337	-	336,192	[56]	✓	×
211	●●Ellagitannin	8.80	785	786	-	618,302,277,251	[57]	×	✓
212	●●Brevifolin	9.30	248	249	-	249,219,191	[42]	×	✓
213	●●Caftaric acid drv.	9.38	668	669	-	312	[29]	✓	✓
214	●●Tetramethyl benzoic acid	10.15	178	179	-	179,150,122	[17]	✓	✓
215	●●Catechin-drv.	10.89	398	399	-	399,290	[30]	×	✓
216	●6-(benzyloxy)-methyl-2,3,4, tri-methyl cyclohexyl formaldehyde	11.58	274	275	-	275	[17]	✓	✓
217	●Allyl pyrocatecol-diacetate	12.0	234	235	-	235	[17]	✓	✓
218	●●Ellagitannin I,II	12.01	644	645	-	301,283,257,193	[44]	✓	×
219	●●9-COA	13.05	398	399	-	399,220,206,179,135	[37]	×	✓
220	●● Iriflophenone-3-C glucoside	13.36	408	409	-	409,273,220,120	[53]	✓	×
221	●●Galloylpyrogallol	13.79	278	279	-	279,153	[29]	✓	×
222	●●Galloylarbutin	15.17	424	425	-	273	[29]	✓	×
223	●●Galloylpyrogalloldrv.	15.49	436	437	-	437,279	([29]	✓	✓
224	●●Methylester of lignoceric acid	19.38	382	383	-	383	[58]	✓	✓
225	●● Methyl corilagin	21.95	648	-	647	649,634,301	[42]	✓	✓
226	●● Dimethyl-corilagin	22.24	662	-	661	661,649,634,301	[42]	✓	✓
227	●●Dihydroisovalerate	30.15	424	425	-	425,365,281	[29]	✓	×

●Compounds previously identified in *Annona muricata*

●●Compounds identified for the first time in *Annona muricata*&annona genus

✓ New compounds identified in *Annona muricata*

HMG= 3-hydroxyl-3-methyl glutary,**CQA**=monocaffeoylquinic acid(chlorogenic acid),**COA**= caffeoyl-2,7-anhydro-2- octulopyranosonic acid

Table S3. Flavonoids and flavonoid derivatives detected and characterized in *A. muricata* in both ethanolic extract of fruit & water extract of the edible part of the fruit by using HPLC–DAD/QTOF-MS in positive and negative ionization modes.

No	Cpd-name	Rt	Mwt	M+	M-	Ms/Ms Fragment	Ref.	E	W
228	✽✽Malonylated-luteolin-*O*-xylose-glucose	3.95	668	669	-	669,537,132	[38]	✓	✓
229	✽✽Apigenin-pentosyl-(hydroxyferuloyl)-pentoside	6.28	726	-	725	533,595,325,,271	[59]	✓	✓
230	✽✽ Luteolin-*O*-caffeoylglucoside	6.72	610	-	609	609,180	[60]	✓	✓
231	✽✽Acacetin-hexose-hexose-glucouronic acid	6.75	784	-	785	785,608,447,338,284, 240,211,176,162	[60]	✓	✓
232	✽✽ Quercetin hexose-malic acid	7.85	580	581	-	463,301	[29]	✓	×
233	✽✽Myricetin-galloyl-pentose	8.26	602	603	-	319,132,171	[61]	✓	×
234	✽✽Myricetin-rhamnose malic acid	8.29	580	581	-	581,463,316,301	[29]	✓	×
235	✽✽ Kaempferol-gallic acid hexose	8.59	600	601	-	601,438,163	[62]	✓	×
236	✽✽Myricetin-galloyl-hexose	9.98	632	633	-	317	[29]	✓	×
237	✽✽Velutin-galloylhexoside	10.11	646	645	-	645,332,314	[29,30]	✓	✓
238	✽✽Delphnidin-*p*-coumaroyl hexose	10.84	611	612	-	611,303	[63]	×	✓
239	✓Dihydromyricetingalloylhexoside	10.92	634	635	-	635, 320, 162, 152	(New Compounds) [30]	✓	×
240	✽✽Dihydroxygallocatechin	11.07	342	-	341	341,305	[64]	✓	✓
241	✓Apigenin-gallate	11.14	422	-	421	421,170,151	(New Compounds) [30]	✓	✓
242	✽ Quercetin-hexoside-rhamnoside-pentose	12.07	742	743		743,308,303	[34]	✓	✓
243	✽✽Dihydrokaempferol-rhamnoside -hexose-pentose	12.25	728	-	727	727,288,294	[34]	×	✓
244	✓Dihydromyricetin-hexouronic acid-hexoside	12.36	658	-	657	657,319,176	(New Compounds) [30]	✓	×
245	✽✽ Kaempferol/luteolin-*O*-pentose-*O*-glucouronic acid	12.40	594	-	593	593,417,285,176	[62,65]	✓	✓
246	✽✽ Delphinidin-3-*p*-coumaroyl-glucose-drv.	**13.00**	727	728	-	728,611,449,278,162, 146,116	[66]	✓	✓
247	✽✽ Malvidin-3-*p*-coumaroyl-glucose-drv.	13.03	785	786	-	639,477,454,308, 162,146	[66]	×	✓
248	✽✽ Delphnidine-3-*O*-(6-*O*-acetyl)-5-*O*-diglucoside	13.15	669	670	-	345	[66]	×	✓
249	✽ Quercetin-pentose-rhamnose	**13.16**	580	581	-	302,265,150	[34]	✓	×
250	✽✽ Naringenin-7-*O*-rutinoside	13.20	580	581	-	271,177,151	[29]	✓	×
251	✽ Rutin(quercetin-rutinoside)	13.35	610	-	609	609,301	[1]	✓	✓
252	✽✽Diosmetin-pentose-glucoside	13.61	594	-	593	593,300	[67]	✓	✓
253	✽✽ Acetyl chrysophanol-O-glucose-xylose	13.62	590	591	-	591,297,253,133	[68]	✓	×
254	✽✽ Apigenin-6-*C*-acetyl-rhamnoside-glucose	13.77	620	621	-	559,455,293	[69]	×	✓
255	✽ Kaempferol/ luteolin-*O*-rutinoside	13.75	594	-	593	593,431,285	[1,34]	✓	✓
256	✽Quercetin-di-glucoside	13.59	626	627	-	627,303	[34]	✓	✓
257	✽✽ Acyl quercetin-rhamnose-glucose	13.81	650	651	-	607,485 ,302,162	[70]	✓	✓
258	✽ Kaempferol-*O*-robinobioside	14.12	594	-	593	593,285	[1]	✓	✓

259	✿✿ Luteolin/kaempferol-diglucoside	14.23	610	-	609	609,285	[60]	✓	✓
260	✿✿ Methyl-kempferol-pentose-hexose	14.43	594	-	593	593,300,285,228	[60]	✓	✓
261	✿✿Naringenin-di-glucoside	14.72	596	597	-	596,505,272,324	[71]	✓	✓
262	✿✿Isorhamntein-pentose-hexose	14.95	610	-	609	609,315,294	[33]	✓	✓
263	✿ Quercetin-3-*O*-neohisperoside	15.05	610	-	609	609,301	[1]	✓	✓
264	✿ Quercetin-3-*O*-robinoside	15.11	610	-	609	609,301	[1]	✓	✓
265	✿✿ Delphnidin-3-*O*-rutinoside	15.21	611	612	-	611,465,303	[62]	×	✓
266	✿✿ Eriodyctoyl-7-*O*-rutinoside	15.60	596	597	-	308,298,163	[38]	✓	✓
267	✿✿ Orientein-7-*O*-deoxy hexose	15.63	594	-	593	594,579,449,,286	[69]	✓	✓
268	✿ Homo-orientin	15.71	448	449	-	448,286,228	[51]	✓	×
269	✿Uercetin-*O*-rhmnoside(quercetrin)	16.13	448	449	-	447,302,146	[48]	✓	×
270	✿Dihydrokaempferol-hexoside	16.15	450	-	449	449,285,162	[1]	✓	×
271	✿ Kaempferol-*O*-hexose	16.43	448	449	-	448,286	[34]	✓	×
272	✿✿ Trihydroxy-6-methoxyflavonone-7-*O*-glucoside	16.44	464	465	-	465,303	[72]	×	✓
273	✿✿Phloretin-*O*-hexoside	16.60	436	437	-	437,275,162	[31]	✓	✓
274	✿✿ Myricetin-3-*O*-rhamnoside	16.61	464	465	-	319,317,300,146	[29]	×	✓
275	✿✿Apigenin-acetyl glucoside	16.63	474	-	473	473,270,203	[73]	×	✓
276	✿✿ Kaempferol / luteolin-rhamnose	16.76	432	-	431	431,287(100%)	[43, 46]	✓	×
277	✿✿Chryoseriol-O-glucoside	16.78	462	463	-	463,301	[38]	✓	✓
278	✿ Quercetin-pentoside	16.78	434	-	433	433,300	[34]	✓	✓
279	✿ Quercetin-glucose/galactose	16.85	464	465	-	302	[1,34]	×	✓
280	✿Dihydrokaempferol-hexoside	16.89	450	-	449	449,287,270	[1]	✓	×
281	✿✿3,3`,7`-trimethyl-sulfate myricetin	17.13	440	441	-	441,318,123	[49]	✓	✓
282	✿✿ Tricetin-4`,-O-glucoside	17.37	464	465	-	303,229,149	[42]	×	✓
283	✿Epi(catechin)	17.50	290	291	-	290,150,136,108	[1,34]	✓	×
284	✿✿Formonontein	17.51	268	269	-	269	[30]	✓	✓
285	✿✿Taxifolin-methylether	17.61	318	319	-	319,257,130	[74]	✓	✓
286	✿✿3`,4`,7- tri-Hydroxy-flavanone	17.77	273	-	272	226,185,158,111	[27]	✓	✓
287	✿✿ Dimethyl quercetin drv.	17.80	616	617	-	617,330	[30]	✓	✓
288	✿Glycitein	17.88	284	-	283	283,266	[51]	✓	✓
289	✿✿ Pinocembrin	17.95	256	-	255	255	[75]	✓	×
290	✿✿Pelargonidin-dimethyldrv.	18.02	301	302	-	302,300	[70]	✓	✓
291	✿✿Liquiritigenin	18.06	256	-	255	255	[75]	✓	×
292	✿Tangeretin	18.57	372	373	-	373,315,300	[17]	✓	×
293	✿Diadzein	18.67	254	255	-	255,237,211,165,145	[51]	✓	×
294	✿Taxifolin	18.69	304	-	303	303,178	[51]	✓	×

295	●●Methoxytaxifolin	18.35	334	-	333	333,303,287(100)	[76]	✓	✓
296	●●Rhamnetin /isorhamnetin	18.65	316	-	315	315,300,284,151	[43]	✓	✓
297	●●Methoxytetrahydroxyisoflavone	18.90	316	-	315	315,272,151	[77]	✓	✓
298	● Kaempferol	18.93	286	287	-	259,229,151	[51]	✓	✓
299	● Luteolin	19.06	286	287	-	269,243,151	[51]	✓	✓
300	●●Myricetin	19.12	318	319	-	319,257,162,102	[29]	✓	✓
301	●Genistein	19.15	270	271	-	271,253,225,215	[51]	✓	✓
302	●●CamellianinA	19.17	620	621	-	433,313	[29]	×	✓
303	●●tri-Hydroxy-methoxy flavone	19.24	300	301	-	301,286	[75]	✓	✓
304	●●tri-Hydroxy-tri-methoxy flavone	19.26	360	361	-	361,343,283,225	[40]	✓	✓
305	●●3,5,7-tri-Methoxy flavone	19.52	312	313	-	313,271,236	[78]	✓	✓
306	●●Chrysoeriol	19.66	300	301	-	301,284,268	[29]	✓	✓
307	●●Methoxykaempferol-methyl ether	21.74	330	331	-	331,285	[56]	✓	✓
308	● Quercetin	23.03	302	303	-	273,229,151	[51]	✓	✓
309	●●Apigenin	23.57	270	271	-	271,253	[75]	✓	✓
310	●Hesperitin	23.71	302	303	-	285,267,231	[51]	✓	✓

● Compounds previously identified in *Annona muricata*

●●Compounds identified for the first time in *Annona muricata* & *Annona genus*

✓New compounds identified in *Annona muricata*

Table S4. Alkaloids detected and characterized in *A. muricata* in both ethanolic extract of fruit & water extract of the edible part of the fruit by using HPLC–DAD/QTOF-MS in positive and negative ionization modes.

No	Cpd-name	Rt	Mwt	M+	M -	Ms/Ms Fragment	Ref.	E	W
311	●2,4,6-tribromoaniline	1.64	329	330	-	298,172,115	[23]	✓	✓
312	●●●Corydine	5.50	341	342	-	342,192	[6]	✓	×
313	●Norcoclaurine	5.52	271	272	-	272,255,240,161	[6]	×	✓
314	●Coclaurine	5.84	285	286	-	269,175,108	[1,6]	✓	×
315	●Reticuline	5.90	329	330	-	330,191	[1,6]	✓	✓
316	●Atherosperminine	6.15	309	310	-	310,295	[1,6]	×	✓
317	●●Actinodaphnine	6.23	311	312	-	312,263,235	[24]	×	✓
318	●Norushinsunine	7.31	281	282	-	282,265	[6]	✓	✓
319	●Anolobine glycoside	7.60	443	442	-	265,247,235,217,162	[6]	✓	×
320	●Annonamine	7.77	296	297	-	297	[1]	✓	✓
321	●Annomuricine	7.85	329	330	-	331	[1]	✓	✓
322	●●●Phytosphinguasine	16.40	317	318	-	317,266,260	[25]	✓	✓
323	●Methylcoclaurine	16.72	299	300	-	300,277	[1]	✓	✓

324	•*N*-acetyl tryptamine	17.66	202	203	-	203,175,161,146,135	[1]	✓	×
325	• Dimethyl coclaurine	17.71	313	314	-	314,300,143,107	[1]	✓	✓
326	•Mangoflorine	18.29	342	-	341	297,282,265,237,222,191	[6]	✓	✓
327	•Coreximine	18.83	327	328	-	328,251	[1,6]	✓	✓
328	•••Corytuberine	18.84	327	328	-	328,297	[6]	✓	✓
329	•Anomurine	18.87	343	-	342	342,175	[1,6]	✓	×
330	•Norcorydine	18.94	327	328	-	328,192	[1,6]	✓	✓
331	•••*Trans*-feruloyl tyramine	19.17	313	314	-	314,178	[6]	✓	✓
332	•••*Trans*-caffeoyl tyramine	19.24	299	300	-	254,163	[6]	✓	✓
333	• DNJ (deoxynojirmycin)<br>•DMJ(deoxymannojirmycin)<br>•DMDP (dideoxy-imino-D-mannitol)	19.22	163	164	-	164	[17]	✓	×
334	•(4-chlorophenyl)-[4-(3-chlorophenyl)2-[(z)-3-(dimethylamino)prop-1-eyl]quinolin-6-yl]-3-methylimmidazol-4-yl) methanol	19.35	543	544	-	543	[23]	✓	×
335	•Annonaine	20.85	266	-	265	266,265,249,219	[1,26]	✓	×
336	•Nuciferine	21.61	295	296	-	297,246,234	[1,26]	×	✓
337	•Xylopine	22.02	295	296	-	296,281,246	[1]	×	✓
338	••Nornuciferine	24.26	281	282	-	282,236,212,174	[6]	✓	✓
339	•Anolobine	24.66	281	282	-	282,265,235	[6]	✓	✓
340	•••Muricinine	24.68	313	314	-	298,163	[6]	✓	✓
341	•••Pronuciferine	25.37	311	312	-	312,266	[6]	✓	✓
342	•Isolaurelrine	26.62	309	310	-	310,279	[1,6]	×	✓
343	••Nordextromethorphan	26.93	257	-	256	256	[17]	✓	✓
344	•••Corydalmine	27.80	341	342	-	342,265	[6]	✓	×
345	•••Corytenchine	28.77	341	342	-	192,165	[6]	✓	×
346	•••Magnoflorinedrv.	28.97	432	-	431	431,342(100%),89	[6]	✓	×
347	•••Dimethylcoclaurinedrv.	30.11	380	381	-	381,313	[1]	✓	✓

• Compounds previously identified in *Annona muricata*
••Compounds identified for the first time in *Annona muricata*
•••Compounds identified for the first time in *Annona* genus

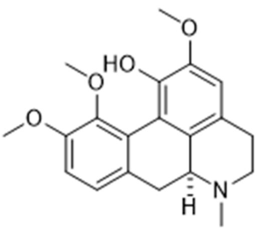

312 Corydine

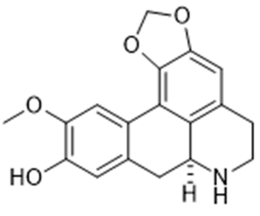

317 Actinodaphnine

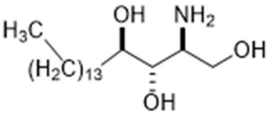

322 Phytosphinguasine

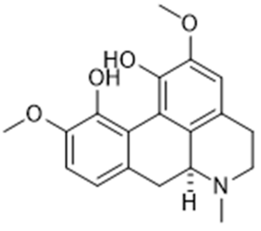

328 Corytuberine

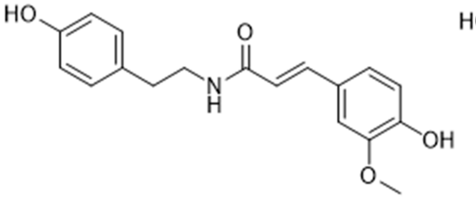

331 *Trans*-feruloyl tyramine

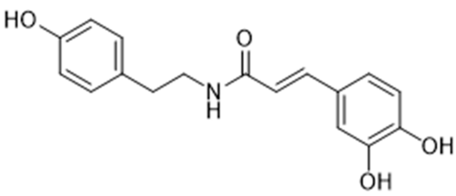

332 *Trans*-caffeoyl tyramine

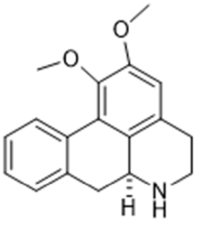

338 Nornuciferine

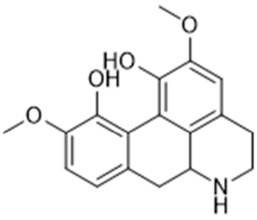

340 Muricinine

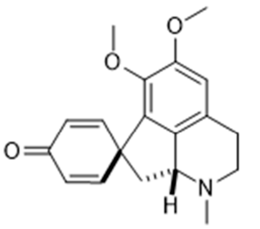

341 Pronuciferine

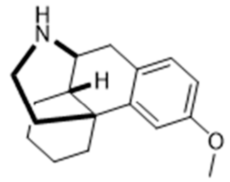

343 Nordextromethorphane

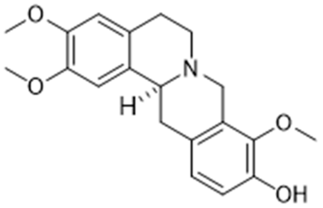

344 Corydalmine

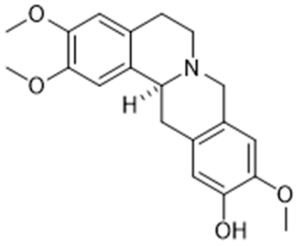

345 Corytenchine

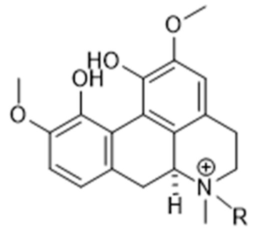

346 Magnoflorine derivative

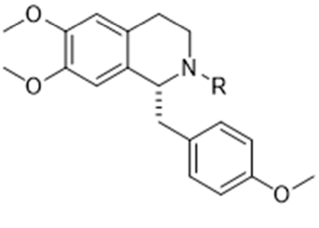

347 Dimethylcoclaurine derivative

Figure S3. Alkaloids identified for the first time from the genus *Annona.*

Table S5. Phytochemical compounds (miscellaneous) detected and characterized in *A. muricata* in both ethanolic extract of fruit & water extract of the edible part of the fruit by using HPLC–DAD/QTOF-MS in positive and negative ionization modes

No	Cpd-name	Type	Rt	Mwt	M+	M -	Ms/Ms Fragment	Ref.	E	W
348	●●● Sucrose	Suger	0.80	342	-	341	179,161,119,113,131	[79]	✓	✓
349	●●●Glucourinoids	Sugers	5.14	630	631	-	547,375,483,146	[38]	✓	×
350	●●● Tyrosine	AA	5.62	181	-	180	180,146,117	[53]	✓	✓
351	●●●Secoisolariresinol	Ph.pro	7.58	362	-	361	362,178	[31]	✓	✓
352	●●7-*O*-methyl oleoresin-pentacetate	Ess.oil	8.62	617	618	-	618,582,516,393,147	[38]	✓	×
353	●●●Coumarin glycoside	Coum	8.98	308	309	-	309,147,162	[30]	✓	×
354	●Triglyceride	FA	9.15	176	177	-	176	[21]	✓	✓
355	●●Esculin-O-glucoside	Coum	9.34	340	341	-	341,178,163	[65]	✓	✓
356	●Mangostin	Xanth.	9.79	410	-	409	409,341,365,326	[16]	✓	×
357	● Aloe emodin	Anthrq	11.12	270	271	-	241,225,211	[51]	✓	✓
358	●●1,1-Dimethyl allyl scopoletin	Coum	12.28	260	261	-	261,161	[17]	✓	×
359	●●●Resverateroldrv.	Coum.	12.36	422	-	421	421,227	[30]	✓	✓
360	●Citroside	MG	13.99	386	-	385	385	[1]	✓	✓
361	●Annoionol B	MG	14.03	244	245	-	245	[1]	✓	✓
362	●Annoionol C	MG	14.25	244	245	-	245	[1]	✓	✓
363	●Rosioside	MG	14.67	386	-	385	385	[1]	✓	✓
364	●Loliolide	MG	16.43	196	197	-	196	[1]	✓	✓
365	●(+)-Epiloliolide	MG	16.81	196	197	-	196	[1]	✓	✓
366	●●●7-demethyl suberosin	Coum	17.52	230	231	-	231,137	[46]	✓	✓
367	●(2)-3-hexenyl-B-D-glucoside	MG	17.54	262	263	-	263	[1]	✓	✓
368	●(1S,2S,4R) trans-2-hydroxy-1,8 cineol-B-D-glucoside	MG	17.83	332	333	-	332	[1]	✓	×
369	●●●Urolithin-B-drv.	Coum	18.58	340	341	-	341,213	[42]	✓	✓
370	●●●Chromonedrv.	Coum	18.70	364	365	-	325	[49]	✓	✓
371	●●●Pimaranediterp	Diterp.	18.84	330	331	-	331,229,205,128	[73]	✓	✓
372	●●●Trijuganone A	Diterp	19.00	294	-	293	293,221,177	[80]	✓	✓
373	●●●15,16-dihydro tanshinone	Diterp.	19.72	278	279	-	170,149	[80]	✓	×
374	●●●1,2-dihydro tanshinone	Diterp.	19.95	278	279	-	200,149	[80]	✓	×
375	●●●2,3,19-tri-OH—urs-12-en-28-oic-acid-glucose	Triterp	22.71	650	651	-	650,503,162	[81]	✓	✓
376	●●●3-oxo-α-ionyl β-d-glucoside	HC	23.73	370	371	-	357,303,185,163	[82]	×	✓
377	● Stigmasterol	Sterol	25.75	412	413	-	413	[21]	✓	✓
378	●β-sitosterol	Sterol	26.03	414	415	-	415	[21]	✓	✓

379	●●2-Chloroethyl lineolate	FA	27.66	342	343	-	342	[17]	✓	✓
380	●●● Linoleic acid methylester	FA	27.73	294	-	293	293,204	[58]	✓	✓
381	●Octadecanoic acid	FA	27.87	284	-	283	283	[16]	✓	✓
382	●●Kaur-16-ene	Sisquit.	29.70	272	273	-	272	[17]	✓	✓
383	●1,3-Tridecanediol diacetate	FA	30.11	300	301	-	301	[17, 45]	✓	✓
384	●●9,10 dehydroisolongifolene	Terpen e	30.33	202	203	-	203 (M+1)	[17]	✓	×
385	●● Oleic acid	FA	30.24	282	-	281	281	[15]	✓	✓
386	●Palmetic acid	FA	30.38	256	-	255	255	[15]	✓	×
387	● 8-heptadecene	HC	30.92	238	239	-	239	[17, 45]	✓	×
388	●●Nonanal	HC	31.24	142	143	-	143	[17]	✓	✓

●Compounds previously identified in *Annona muricata*
●●Compounds identified for the first time in *Annona muricata*
●●●Compounds identified for the first time in *Annona* genus

Alks: alkaloids; Coum: coumarins; Diterp: diterpenoids; FA: fatty acids; HC: hydrocarbon; Mg: megastimane; Sesquit: sesquiterpene; Triterp: triterpene; Xanth: xanthene; E: Ethanol extract; W: Water extract.

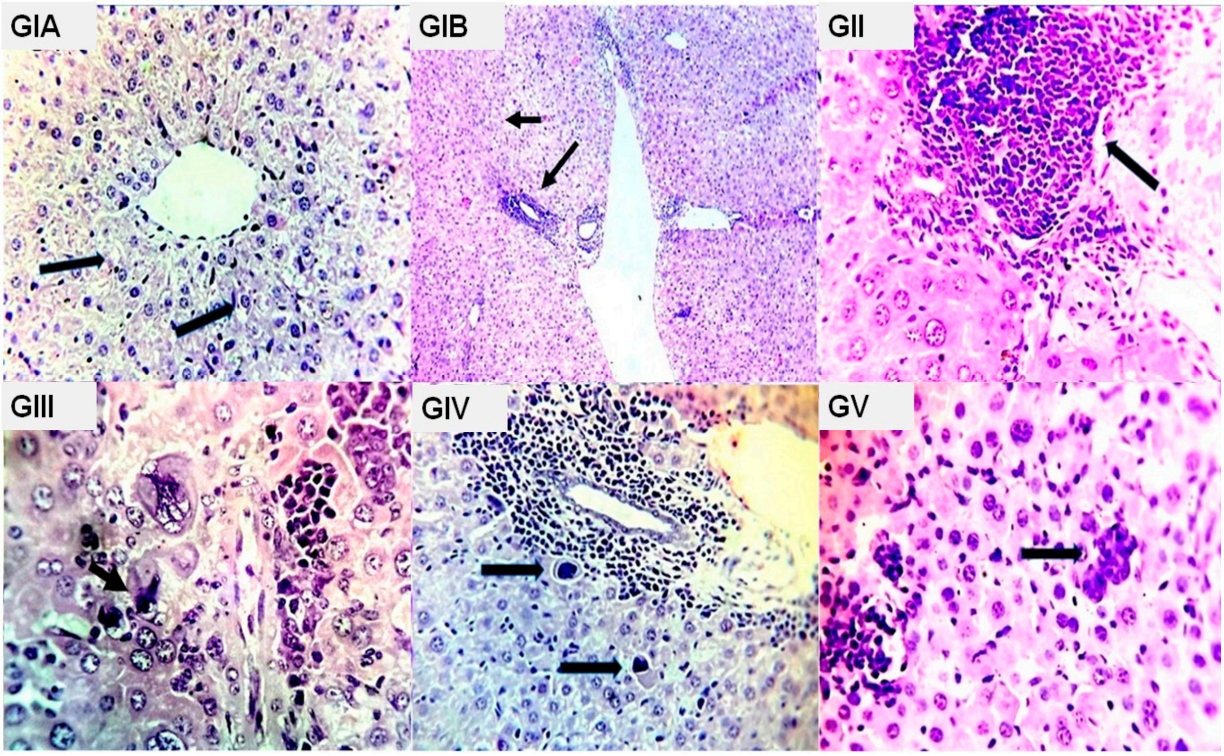

Figure S4. Photomicrograph from the liver of different experimental groups (**GII-GV**). **GI**: Normal hepatic parenchyma with preserved portal triades and keeping features of the hepatocytes, portal blood vessels, sinusoids, and von-kupffer cells (black arrows). **GII**: Infiltration by abnormal epithelial tumor cells with ovoid large hyperchromatic nuclei, they appear adjacent to the portal triades and partially replacing the hepatic parenchyma (black arrow). **GIII**: Remnants of degenerated tumor cells (black arrow) destructed by aggressive inflammatory reaction of lymphocytes and macrophage cells. **GIV**: Revealing very few numbers of distributed viable atypical epithelial tumor cells with very few mitotic activities (black arrow). **GV**: Denoted tiny small aggregates of atypical tumor cells (black arrow) some are surrounding by large number of immune cells including lymphocytes and plasma cells.

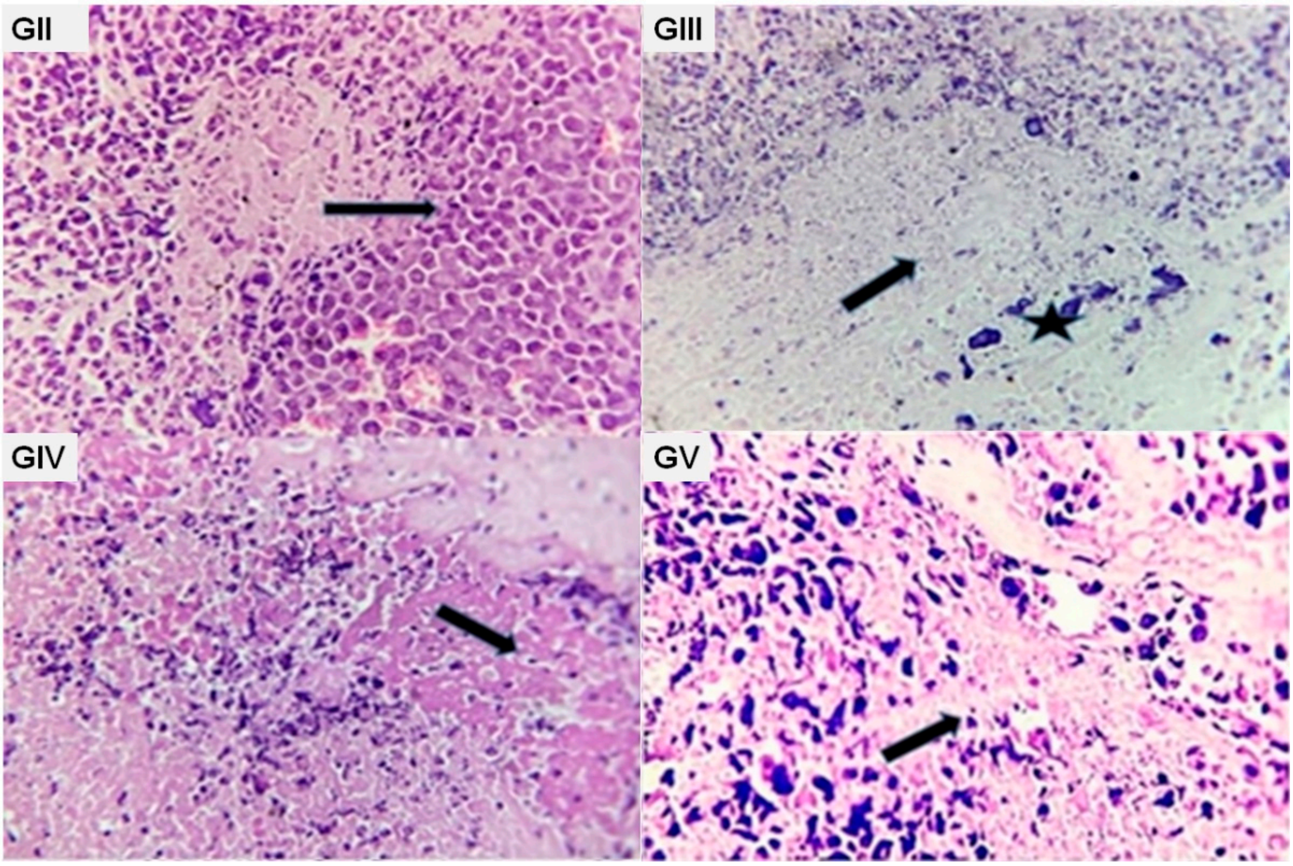

Figure S5. Photomicrograph of tumor mass of different experimental groups (**GII-GV**). **GII**: The tumor cells showing moderate mitotic activity (black arrow); **GIII**: The tumor mass with complete necrotic changes (black arrow) with focal calcification (black star); **GIV**: The tumor mass appears with necrotic and apoptotic changes in 80%-85% of the tumor cells (black arrow); **GV**: Completely necrotic intraperitoneal tumor mass with massive tissue necrosis and replacement by inflammatory cells (black arrow).

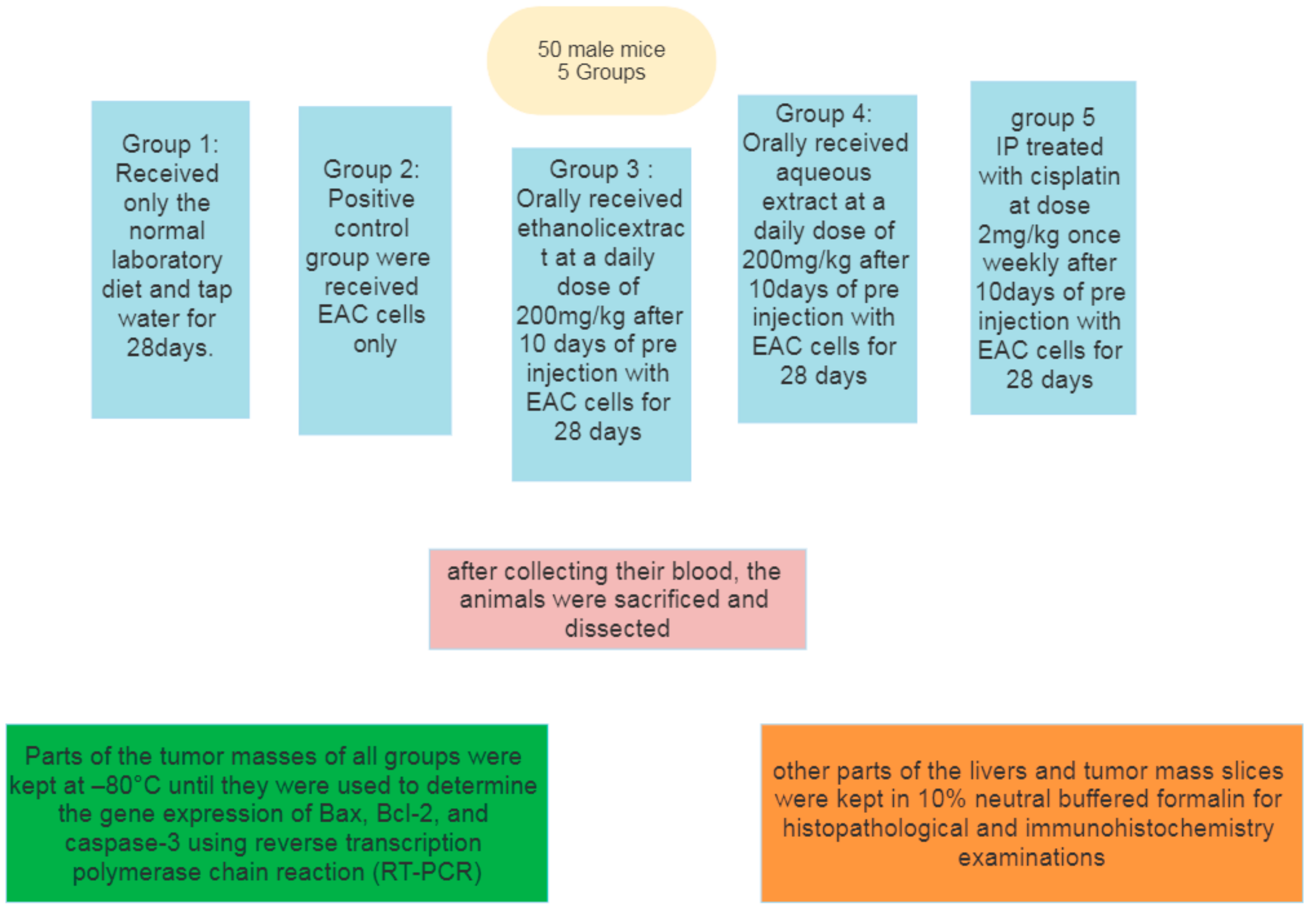

Figure S6. Flow chart of animals' cytotoxic study
